# Impact of DLK1-DIO3 imprinted cluster hypomethylation in smoker patients with lung cancer

**DOI:** 10.18632/oncotarget.10611

**Published:** 2016-07-15

**Authors:** Sonia Molina-Pinelo, Ana Salinas, Nicolás Moreno-Mata, Irene Ferrer, Rocío Suarez, Eduardo Andrés-León, Manuel Rodríguez-Paredes, Julian Gutekunst, Eloisa Jantus-Lewintre, Carlos Camps, Amancio Carnero, Luis Paz-Ares

**Affiliations:** ^1^ Instituto de Biomedicina de Sevilla (IBIS) (HUVR, CSIC, Universidad de Sevilla), Sevilla, Spain; ^2^ Medical Oncology Department, Hospital Universitario Doce de Octubre & Centro Nacional de Investigaciones Oncológicas (CNIO), Madrid, Spain; ^3^ CIBER de Cáncer, Madrid, Spain; ^4^ Thoracic Surgery Department, Hospital Universitario Virgen del Rocio, Sevilla, Spain; ^5^ Division of Epigenetics, DKFZ-ZMBH Alliance, German Cancer Research Center, Heidelberg, Germany; ^6^ University Tumor Center Düsseldorf, University of Düsseldorf, Düsseldorf, Germany; ^7^ Molecular Oncology Laboratory, Fundación para la Investigación del Hospital General Universitario de Valencia, Valencia, Spain; ^8^ Department of Biotechnology, Universitat Politècnica de Valencia, Valencia, Spain; ^9^ Department of Medicine, University of Valencia, Valencia, Spain; ^10^ Department of Medical Oncology, Hospital General Universitario de Valencia, Valencia, Spain

**Keywords:** lung cancer, epigenetic, *DLK1-DIO3* cluster, transcriptional regulation, COPD

## Abstract

DNA methylation is important for gene expression and genome stability, and its disruption is thought to play a key role in the initiation and progression of cancer and other diseases. The *DLK1-DIO3* cluster has been shown to be imprinted in humans, and some of its components are relevant to diverse pathological processes. The purpose of this study was to assess the methylation patterns of the *DLK1-DIO3* cluster in patients with lung cancer to study its relevance in the pathogenesis of this disease. We found a characteristic methylation pattern of this cluster in smoking associated lung cancer, as compared to normal lung tissue. This methylation profile is not patent however in lung cancer of never smokers nor in lung tissue of COPD patients. We found 3 deregulated protein-coding genes at this locus: one was hypermethylated (*DIO3*) and two were hypomethylated (*DLK1* and *RTL1*). Statistically significant differences were also detected in two different families of SNORDs, two miRNA clusters and four lncRNAs (*MEG3*, *MEG8*, *MEG9* and *LINC00524*). These findings were validated using data from the cancer genome atlas (TCGA) database. We have then showed an inverse correlation between DNA methylation and expression levels in 5 randomly selected genes. Several targets of miRNAs included in the *DLK1-DIO3* cluster have been experimentally verified as tumor suppressors. All of these results suggest that the dysmethylation of the imprinted *DLK1-DIO3* cluster could have a relevant role in the pathogenesis of lung cancer in current and former smokers and may be used for diagnostic and/or therapeutic purposes.

## INTRODUCTION

Lung cancer is a highly incident disease, commonly associated to tobacco exposure, and currently represents the most frequent cause of cancer mortality. More than 80% of the cases present non-small cell lung cancer (NSCLC) histology, including adenocarcinoma and squamous cell carcinoma [1, 2]. Many molecular analyses have shown the complexity driving the carcinogenesis of the clinically relevant lung cancer phenotypes [3]. This includes DNA methylation patterns that undergo complex changes in cancer [4]. This process is important for gene expression and genome stability, and its disruption is thought to play a key role in the initiation and progression of lung cancer [5].

According to classical Mendelian laws, the majority of genes in a human cell are inherited in two functionally equivalent parental copies. However, there is a small subset of genes with one turned-off copy in a parent-of-origin-dependent manner[6]. This phenomenon, known as genomic imprinting, is an epigenetic process that involves monoallelic expression. Some genes are imprinted across the complete genome sequence, but the vast majority of imprinted genes are clustered [7, 8]. Genomic imprinting occurs in many diseases, such as neurological disorders, cancer and many others [9–11]. Loss of imprinting is commonly observed in human tumors [12], suggesting the presence of the imbalanced expression of imprinted tumor-suppressor genes or oncogenes. The *IGF2* and *P73* genes have been pointed out as examples of imprinted oncogene and tumor suppressor gene, respectively, in several tumors [11, 13–20].

The *DLK1-DIO3* imprinted cluster is located on chromosome 14q32.2. This region includes protein-coding genes (*DLK1, RTL1* and *DIO3*), long non-coding RNAs (*MEG3, MEG8, MEG9* and *LINC00524*), two large clusters of miRNAs, two families of small nucleolar RNAs (*SNORD113* and *SNORD114*) and several pseudogenes [21]. Aberrations involving some components of the *DLK1-DIO3* cluster have been linked to pathological processes [22]. Increased *DLK1* levels may become oncogenic in NSCLC by upregulating cell cycle machinery, which has been proposed to be highly associated with tumor invasion [23]. Similarly, overexpression of the *RTL1* gene promotes hepatocarcinogenesis [24]. miRNAs located in the DLK1-DIO3 cluster have been shown to be involved in the development of different tumors such as lymphomas, glioblastomas, gastric tumors, and many others [22]. At the same time, it has been demonstrated that *SNORD113* expression is linked to decreased survival in patients with hepatocellular carcinoma [25]. Concerning lung cancer, Valdmanis *et al*. recently described that the expression of a cluster of ∼53 microRNAs and mRNAs at the *DLK1-DIO3* locus on mouse chromosome 12qF1 was markedly increased in tumors compared to non-tumoral tissue, and they have proposed a role for the cluster in the origin of cancer cells [26]. Altogether, the aforementioned data suggest aberrant epigenetic regulation in genomic imprinting may promote imbalanced growth, thus leading to human tumorigenesis. To elucidate the role of the *DLK1-DIO3* cluster in the tumorigenesis of non-small cell lung cancer, we have analyzed the methylation pattern in lung tumors as compared to non-tumoral lung tissue.

## RESULTS

### DNA methylation pattern of the *DLK1-DIO3* cluster in lung cancer

**Figure 1:**
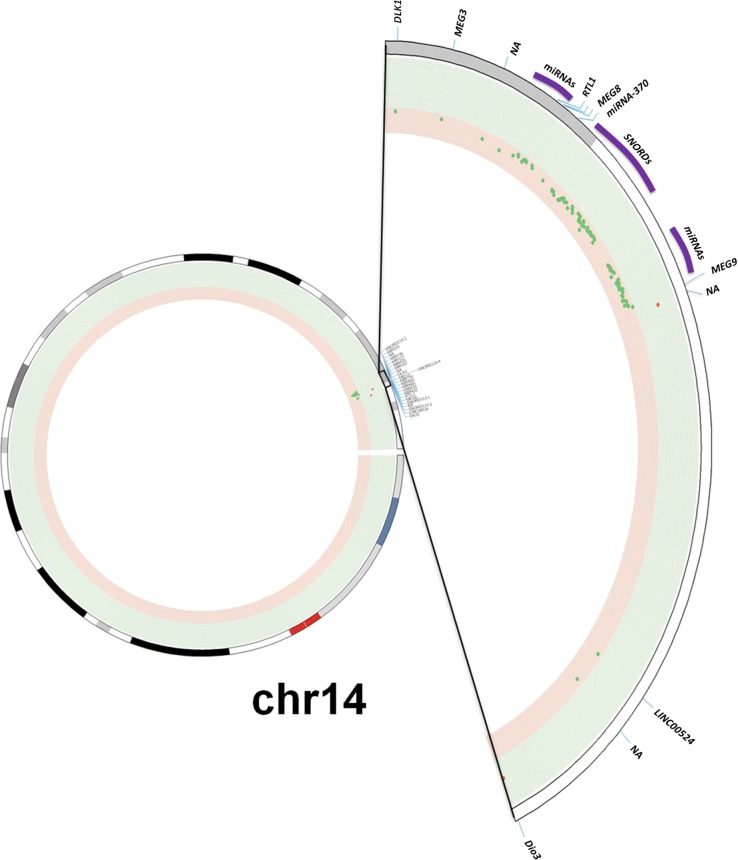

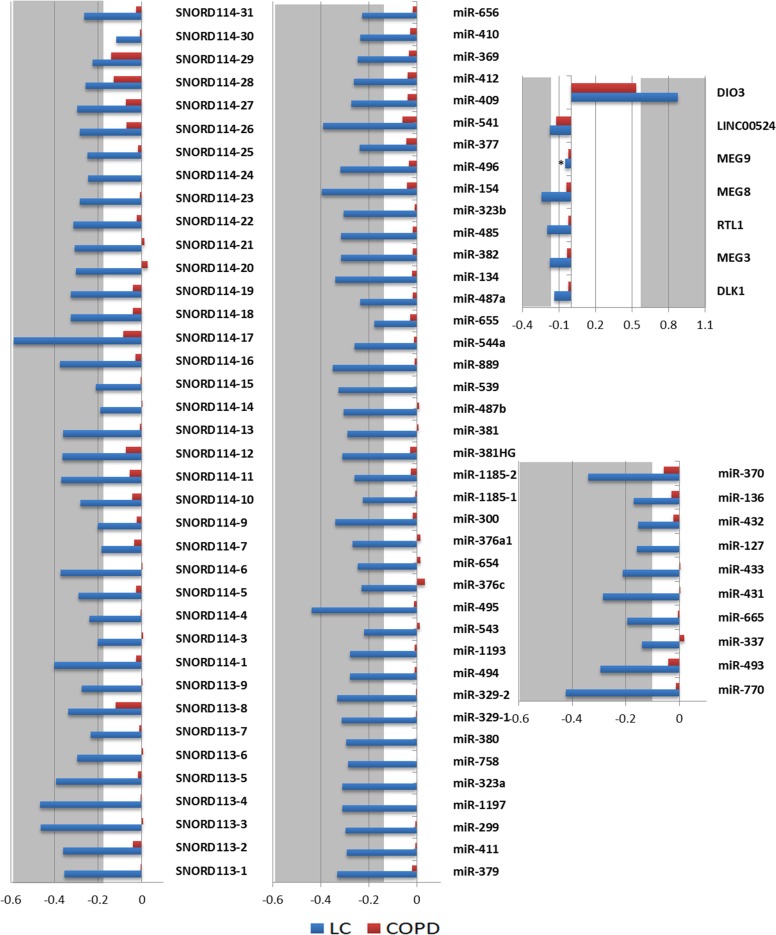
Methylation profile of the *DLK1-DIO3* cluster in lung cancer **A.** The *DLK1-DIO3* cluster is located on chromosome 14 (14q32). Using a Circos plot, we plotted the methylation levels on chromosome 14. From inside to outside: methylation levels, ideogram and gene labels. Hypermethylation (red dots and green background) and hypomethylation events (green dots and red background) in patients with lung cancer versus non-tumoral samples. **B.** Observed methylation changes (log_2_ ratio). Relative levels of methylation in patients with lung cancer relative to the control group are represented in blue bars, whereas methylation levels of COPD patients with respect to the control group are represented by red bars. A grey background or asterisks represent statistically significant differences (adjusted p-value < 0.05) of methylation levels with respect to the control group. NA: Not available.

**Figure 2:**
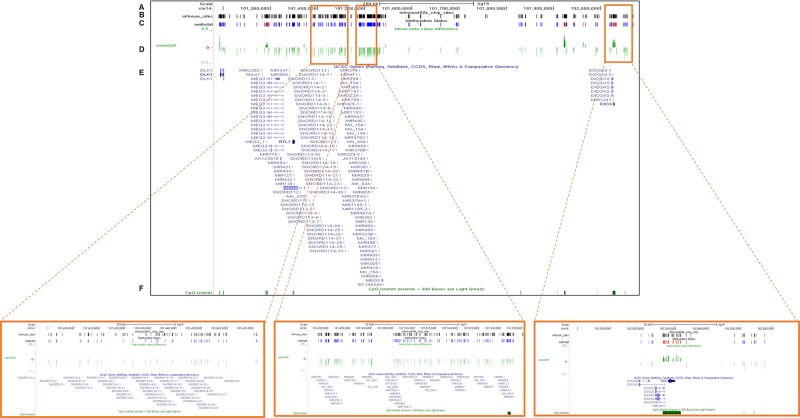
Validation of the DNA methylation status of the *DLK1-DIO3* cluster using TCGA data The plot shows the methylation analysis of the *DLK1-DIO3* cluster in lung cancer versus non-tumoral tissue (100 vs. 32 samples) of current and former smokers visualized by UCSC browser. Zoomed-in views of the *SNORD114-9, miR-889* and *DIO3* regions are highlighted in orange squares. **A.** Chromosome 14-scale. **B.** CpG sites included in the Illumina Infinium Human Methylation 450 BeadChip. **C.** Differential DNA methylation in lung cancer versus non-tumoral tissue of current and former smokers. Bars represent hypermethylated (red) and hypomethylated (blue) probes in lung cancer tissue with respect to normal tissue. **D.** ß-value differences between lung cancer and non-tumoral tissue. **E.** Gene names of the members of the *DLK1-DIO3* cluster, and **F.** the position of the CpG islands present in the *DLK1-DIO3* cluster (green).

**Figure 3:**
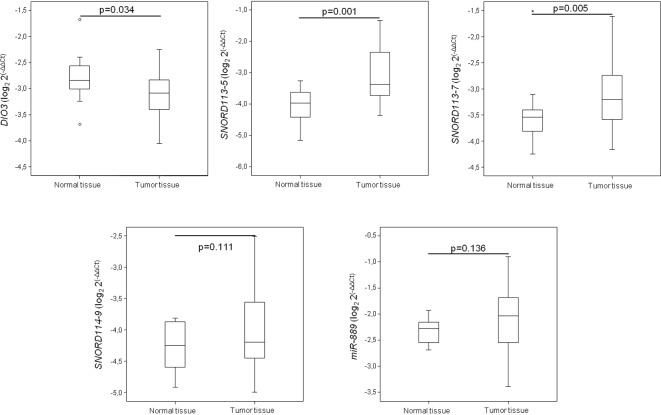
mRNA expression levels of some *DLK1-DIO3* cluster components Expression of some *DLK1-DIO3* cluster components, which was validated by real-time PCR. mRNA expression levels were determined in tumor samples and paired normal lung tissue from lung cancer patients. Data derived from RT-qPCR are presented as the log_2_ 2^-ΔΔCt^ values.

To evaluate the potential role of the *DLK1-DIO3* cluster in lung cancer, we analyzed the methylation status of the cluster in human lung tissue. The methylation profile of *DLK1-DIO3* was evaluated in human tumor samples and compared to non-tumoral tissue using the Illumina Infinium Human Methylation 450 BeadChip. The methylation levels in lung cancer versus non-tumoral tissue are represented in Figure 1A and Supplementary Table S1. Patients with lung cancer showed three deregulated protein-coding genes: one was hypermethylated (*DIO3*) and two were hypomethylated (*DLK1* and *RTL1*). Statistically significant differences (adjusted p-value < 0.05) were also detected in two different families of SNORDs (*SNORD113* and *SNORD114*), with the exception of *SNORD114-30* that showed a similar but non-significant pattern of DNA hypomethylation (p=0.07), (Table 1). Similarly, two miRNA clusters and four lncRNAs (*MEG3, MEG8, MEG9* and *LINC00524*) were found to be significantly less methylated in tumors as compared to non-tumoral tissue (Figure 1B and Table 1). To validate these findings we used methylation data from an independent cohort obtained from TCGA database, and a different method of analysis. Consistently significant differences in methylation were observed again for the *DLK1-DIO3* cluster (Figure 2). All genes included in the cluster show a hypomethylated pattern with the exception of *DIO3* that was hypermethylated in tumor tissue. In order to investigate methylation-dependent RNA expression changes, some components of the *DLK1-DIO3* cluster, such as *DIO3*, *SNORD113-5*, *SNORD113-7*, *SNORD114-9*, and *miR*-889, were randomly selected to analyze their expression levels in matched normal-tumor samples. The results confirmed that the methylation levels were inversely associated with the expression levels, i.e., hypomethylation of *SNORD113-5*, *SNORD113-7*, *SNORD114-9*, and *miR*-889 correlated with high gene expression in tumor samples relative to non-tumoral tissue. In the case of *DIO3*, we found lower levels of mRNA expression in lung tumor tissue (Figure 3).

**Table 1:** Statistical differences between miRNAs and SNORDs in human tumor samples compared to non-tumoral tissue

	Genes	Adjusted p-value
**miRNAs**	*miR-770*	*2.51232E-14*
	*miR-493*	*5.81898E-11*
	*miR-337*	*7.41923E-06*
	*miR-665*	*1.16877E-08*
	*miR-431*	*7.32285E-13*
	*miR-433*	*2.10219E-11*
	*miR-127*	*1.32018E-08*
	*miR-432*	*2.10017E-08*
	*miR-136*	*6.70757E-09*
	*miR-370*	*6.16842E-11*
	*miR-379*	*2.90118E-17*
	*miR-411*	*3.10121E-17*
	*miR-299*	*7.69421E-17*
	*miR-1197*	*1.37059E-15*
	*miR-323A*	*1.73804E-15*
	*miR-758*	*1.45179E-14*
	*miR-380*	*1.13522E-14*
	*miR-329-1*	*6.75807E-14*
	*miR-329-2*	*1.64162E-13*
	*miR-494*	*3.65725E-12*
	*miR-1193*	*3.65725E-12*
	*miR-543*	*9.62007E-09*
	*miR-495*	*6.14013E-16*
	*miR-376C*	*2.39664E-11*
	*miR-654*	*3.35623E-12*
	*miR-376A1*	*8.48877E-13*
	*miR-300*	*1.65112E-14*
	*miR-1185-1*	*1.86087E-11*
	*miR-1185-2*	*6.87418E-14*
	*miR-381HG*	*1.17148E-14*
	*miR-381*	*7.43382E-11*
	*miR-487B*	*3.24504E-12*
	*miR-539*	*4.29749E-14*
	*miR-889*	*2.92232E-16*
	*miR-544A*	*1.15212E-12*
	*miR-655*	*1.48893E-07*
	*miR-487A*	*8.934E-13*
	*miR-382*	*1.90249E-14*
	*miR-485*	*1.90249E-14*
	*miR-323B*	*1.05772E-13*
	*miR-154*	*3.35275E-17*
	*miR-496*	*2.18282E-14*
	*miR-377*	*1.07483E-09*
	*miR-541*	*3.62941E-15*
	*miR-409*	*1.59813E-13*
	*miR-412*	*4.93041E-13*
	*miR-369*	*2.73461E-12*
	*miR-410*	*9.88918E-12*
	*miR-656*	*5.71628E-12*
**SNORDs**	*SNORD113-1*	*2.85848E-13*
	*SNORD113-2*	*1.06116E-08*
	*SNORD113-3*	*2.81624E-08*
	*SNORD113-4*	*8.3473E-11*
	*SNORD113-5*	*1.37034E-10*
	*SNORD113-6*	*5.34803E-09*
	*SNORD113-7*	*0.003019259*
	*SNORD113-8*	*1.63653E-07*
	*SNORD113-9*	*1.80412E-09*
	*SNORD114-1*	*5.89536E-14*
	*SNORD114-3*	*0.001522221*
	*SNORD114-4*	*7.88509E-06*
	*SNORD114-5*	*1.21425E-08*
	*SNORD114-6*	*4.64279E-06*
	*SNORD114-7*	*9.64877E-06*
	*SNORD114-9*	*0.000312165*
	*SNORD114-10*	*6.11146E-06*
	*SNORD114-11*	*4.40691E-09*
	*SNORD114-12*	*1.6391E-09*
	*SNORD114-13*	*3.02053E-09*
	*SNORD114-14*	*1.02738E-07*
	*SNORD114-15*	*1.36835E-07*
	*SNORD114-16*	*1.36239E-10*
	*SNORD114-17*	*8.38123E-14*
	*SNORD114-18*	*6.61886E-14*
	*SNORD114-19*	*6.61886E-14*
	*SNORD114-20*	*0.009700679*
	*SNORD114-21*	*1.49467E-07*
	*SNORD114-22*	*1.06972E-08*
	*SNORD114-23*	*1.45598E-08*
	*SNORD114-24*	*8.99314E-10*
	*SNORD114-25*	*4.74915E-08*
	*SNORD114-26*	*2.01146E-06*
	*SNORD114-27*	*6.35641E-09*
	*SNORD114-28*	*2.50354E-07*
	*SNORD114-29*	*9.6575E-05*
	*SNORD114-30*	0.071177541
	*SNORD114-31*	*9.91748E-07*
	*SNORD114-31*	*9.91748E-07*
	*SNORD114-31*	*9.91748E-07*

### *DLK1-DIO3* methylation profile by smoking characteristics

**Figure 4:**
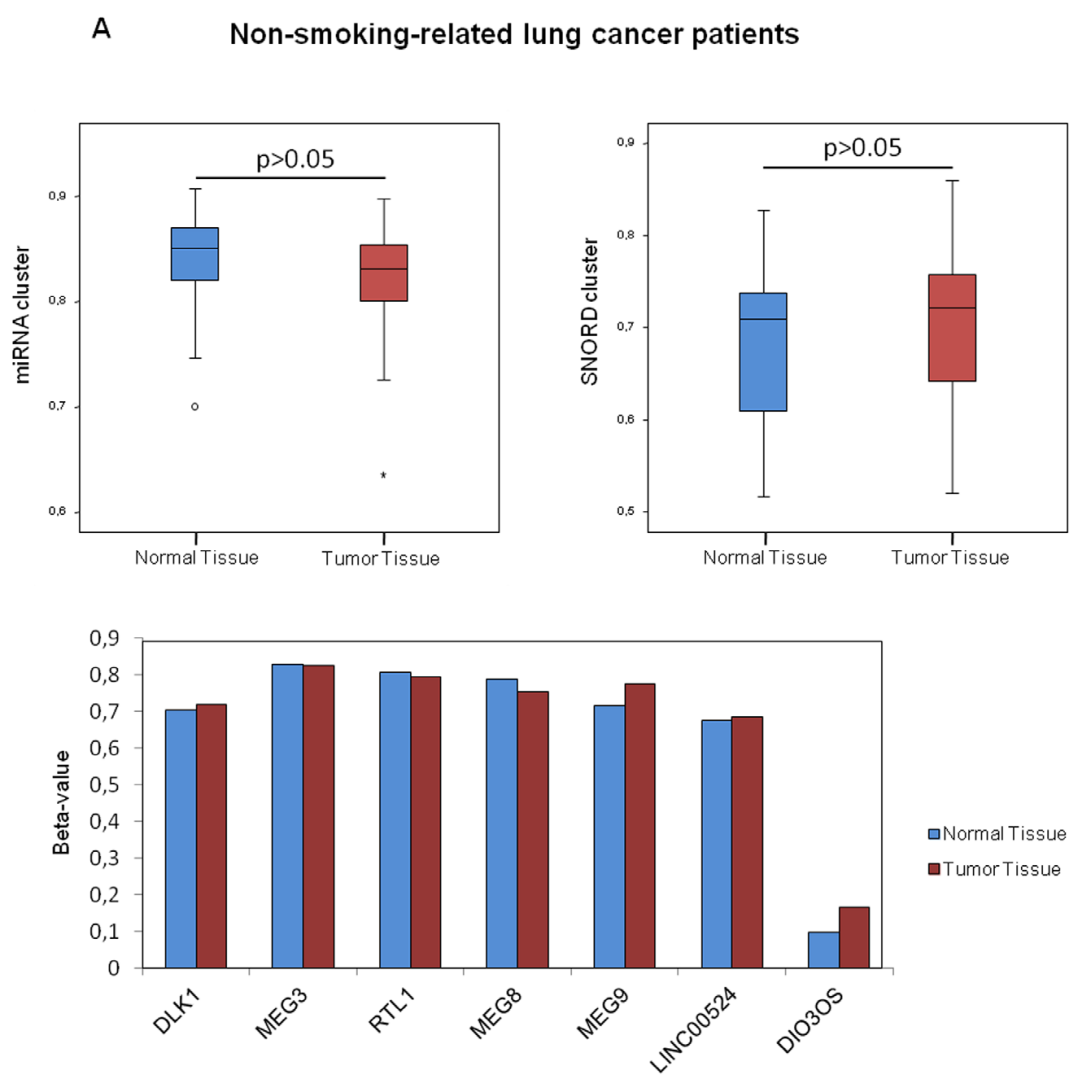

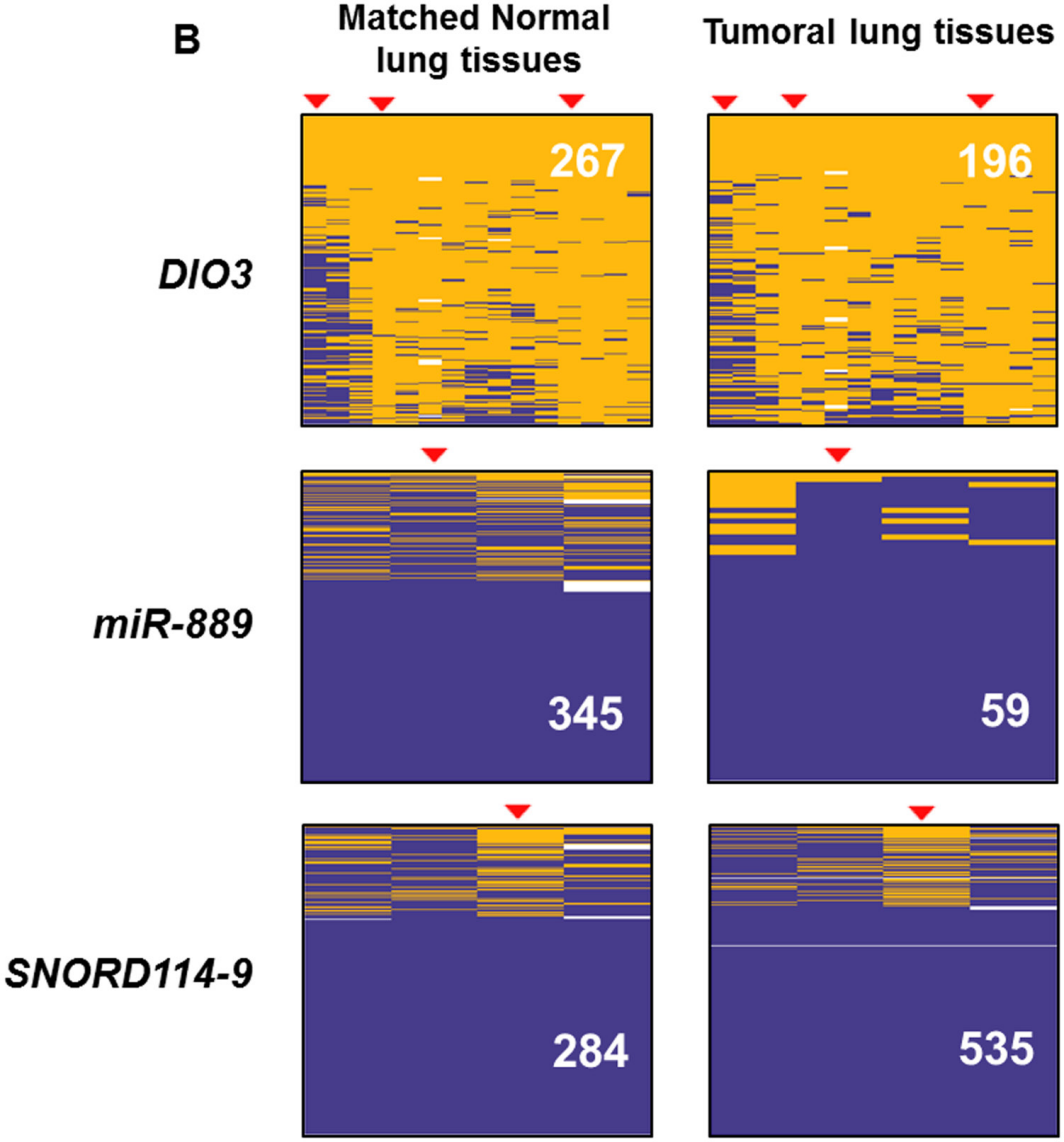

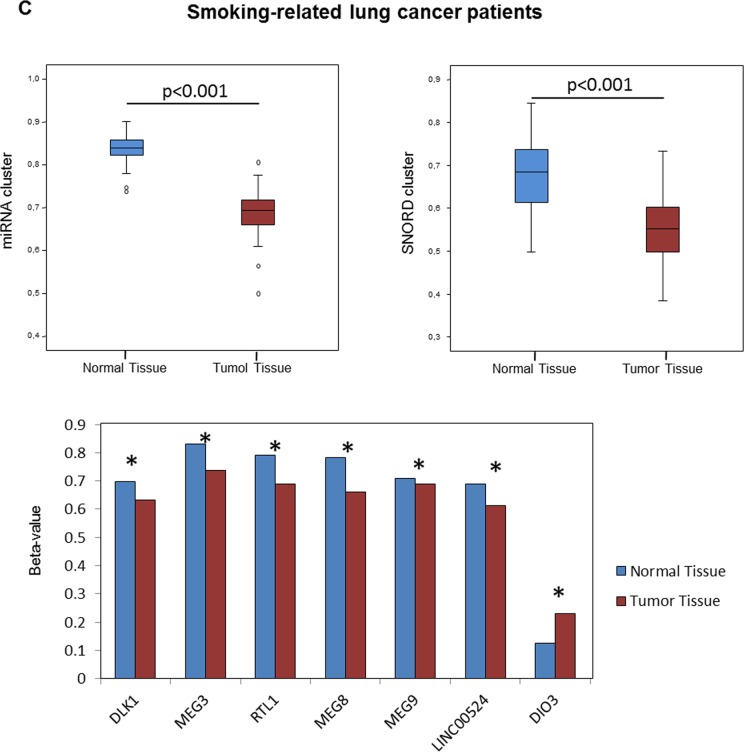
Association between smoking behavior and methylation profile of the *DLK1-DIO3* cluster **A.** *DLK1-DIO3* cluster methylation level differences between non-tumoral and tumoral tissue in non-smoking-related lung cancer patients. **B.** Validation of the DNA methylation status of *DIO3, miR-889* and *SNORD114-9* by 454 bisulfite sequencing using pools of 10 non-tumoral and tumoral tissue samples in non-smoking-related lung cancer patients. Each column shows the methylation status of an individual CpG and the rows (and also the white numbers) indicate the amount of reads. The red arrowheads highlight the position of the CpG represented on the Illumina Infinium Human Methylation 450 BeadChip. Methylated CpGs are displayed in purple, unmethylated CpGs in orange and gaps in white. **C.** *DLK1-DIO3* cluster methylation level differences between normal tissue and tumoral tissue in smoking-related lung cancer patients, including current and former smokers. ^*^: adjusted p-value<0.001. β-value: sum of methylated and unmethylated probe intensities.

We evaluated variations of the *DLK1-DIO3* methylation profile of lung tissues, tumoral and non-tumoral, according to the prior smoking exposure of patients. We found no differences on cluster *DLK1-DIO3* methylation between non-tumoral and tumoral lung tissue among never smokers (Figure 4A). The methylation status of *DIO3, miR-889* and *SNORD114-9* was further validated by 454 bisulfite sequencing using pooled non-tumor and tumor samples from non-smokers (Figure 4B). In current and former smokers with lung cancer, the *DLK1-DIO3* cluster showed higher methylation in non-tumoral tissue as compared to tumor. In the latter group of patients, statistically significant differences in methylation were identified in all components of the *DLK1-DIO3* cluster (adjusted p-value <0.001) (Figure 4C).

We then next analyzed whether the DNA methylation pattern in non-tumoral lung parenchyma of current and former smokers with COPD differed from that of current and former smokers without COPD and found no statistical differences (Figure 1B). Of note, an increase in the DNA promoter methylation of *DIO3* was noticeable in COPD lung tissue although not statistically significant (log_2_ ratio COPD^+^/COPD^-^LC^-^ was 0.535 and the adjusted p-value was 0.840) (Supplementary Table S1). In addition, we have found that the *DLK1-DIO3* methylation profile of non-malignant tissue from lung cancer patients and control subjects was similar (data not shown).

### *DLK1-DIO3* methylation profile by histological subtypes

**Figure 5:**
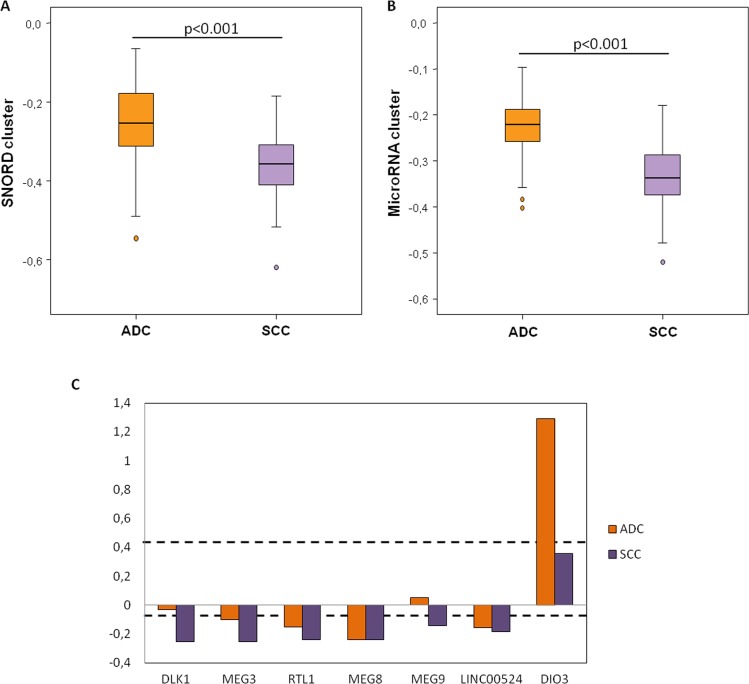
Methylation levels of the *DLK1-DIO3* cluster components according to the histological subtype of lung cancer **A.** Relative SNORD methylation levels in patients with squamous cell carcinoma (SCC) relative to the control group is represented in purple bars, whereas methylation levels in patients with adenocarcinoma (ADC) with respect to the control group are represented by orange bars. **B.** Relative microRNA methylation levels in patients with SCC (purple) and ADC (orange) relative to the control group. **C.** Relative methylation levels of *DIO3, LINC00524, MEG9, MEG8, RTL1, MEG3* and *DLK1* in patients with SCC (purple) and ADC (orange) with respect to the control group. Black broken lines represent statistically significant differences (adjusted p-value < 0.05) of methylation levels with respect to the control group.

Due to its specific global hypomethylation in lung cancer, we have evaluated the methylation of the *DLK1-DIO3* cluster in the two main histological subtypes of NSCLC, squamous cell carcinoma (SCC) and adenocarcinoma, as compared to non-tumoral tissue. The *DLK1-DIO3* imprinted cluster hypomethylation was consistently evident in both histological subtypes with respect to non-tumoral tissue (Supplementary Table S1). We found that SCC samples showed significantly higher hypomethylation levels in the SNORD cluster than in lung adenocarcinoma (Figure 5A). As with *SNORD114-30* for general lung cancer, we did not find any statistically significant differences for lung adenocarcinoma or SCC (adjusted p-value=0.494 and 0.095, respectively). *SNORD113-7* and *SNORD114-3* were only statistically significant in the SCC group (log_2_ ratio SCC^+^/non-tumoral tissue = -0.309; adjusted p-value= 0.025 and log_2_ ratio SCC^+^/non-tumoral tissue= -0.234; adjusted p-value = 0.031, respectively) (Supplementary Table S1). MicroRNA clusters were also notably more hypomethylated in SCC (Figure 5B). *RTL1, MEG8* and *LINC00524* were hypomethylated in both histological subgroups. Moreover, *DLK1* and *MEG9* were significantly hypomethylated in patients with SCC (p<0.001 and p=0.01, respectively), while in lung adenocarcinoma, *DLK1* was very similar to the control group (p=0.127). *DIO3* was significantly hypermethylated in patients with lung adenocarcinoma (p<0.001) (Figure 5C).

### Transcriptional mapping of the *DLK1-DIO3* cluster

**Figure 6:**
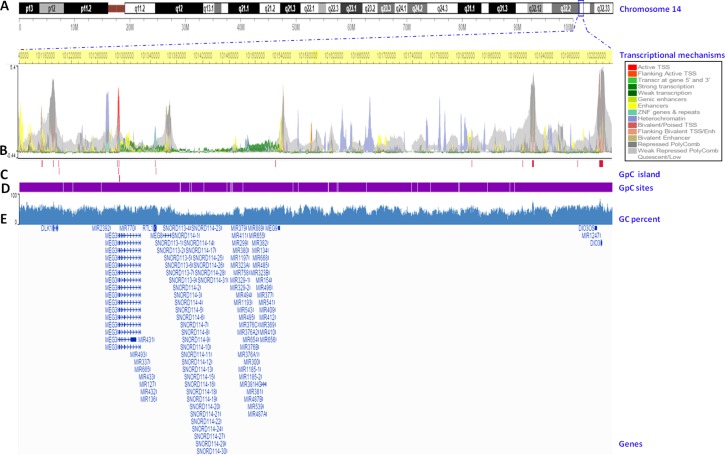
Transcriptional mapping of the *DLK1-DIO3* cluster to the human reference genome hg19 **A.** Chromosome 14 ideogram. The *DLK1-DIO3* cluster is located on 14q32. The exact position of the cluster in the region is marked with a blue square. **B.** Chromosome position. Base pairs of the *DLK1-DIO3* cluster on chromosome 14 (highlighted in yellow). The transcriptional mechanisms underlying the expression of the *DLK1-DIO3* cluster, such as the transcription start sites (TSS), enhancer regions (Enh), zinc finger (ZNF), packed form of DNA, and polycomb group proteins. **C.** CpG islands in the *DLK1-DIO3* cluster (crimson lines). **D.** The genomic distribution of CpG sites in the *DLK1-DIO3* cluster (white lines). **E.** GC percent in the *DLK1-DIO3* cluster (blue). Finally, the sequence references of members of the *DLK1-DIO3* cluster are represented.

To analyze genomic features associated with different mechanisms of the transcriptional regulation of the *DLK1-DIO3* cluster, we used the Wash U Epigenome Browser to display the epigenomic mapping of the *DLK1-DIO3* cluster (14q32) to the human genome reference (*hg19*; chr14: 101.140.120–102.044.779 genomic coordinates) (Figure 6A). We observed the distribution of several transcriptional mechanisms (direct regulators and structural determinants) along the *DLK1-DIO3* cluster (Figure 6B). Multiple enhancers are distributed in the length of the cluster (yellow arrows), with the exception of the region located between 101380-10530Kb on chromosome 14. The *SNORD113* and *SNORD114* families and a miRNA cluster are located in this region without enhancers. In this same region, we found few effects of polycomb protein-mediated epigenetic regulation (grey arrows). In addition, we identified a heterochromatin rich domain situated approximately 101277Kb distal to the 5’ region (purple arrows). Therefore, following with *MEG9* lncRNA (101536Kb), we found a highly condensed region before it reached the 5′ region of the *DIO3* gene. Interestingly, the 5’ flanking region of the *MEG3* lncRNA (∼101292 kb) showed activity in transcription initiation, where there is an active transcriptional start site (TSS) marked by a red arrow. No other region of the *DLK1-DIO3* cluster showed active TSS. From the active TSS, we found an approximately 240Kb a domain with strong transcription (green arrows) (Figure 6B). However, we identified several CpG islands located near genes throughout this region, including lncRNAs, miRNAs, SNORDs and pseudogenes (crimson lines) (Figure 6C). However, the CpG sites were distributed along the *DLK1-DIO3* cluster (white lines) (Figure 6D). The GC-content was reduced by 30% from *MEG3* to *MEG9* (Figure 6E).

### Experimentally validated target interactions of the *DLK1-DIO3* miRNA cluster

**Figure 7:**
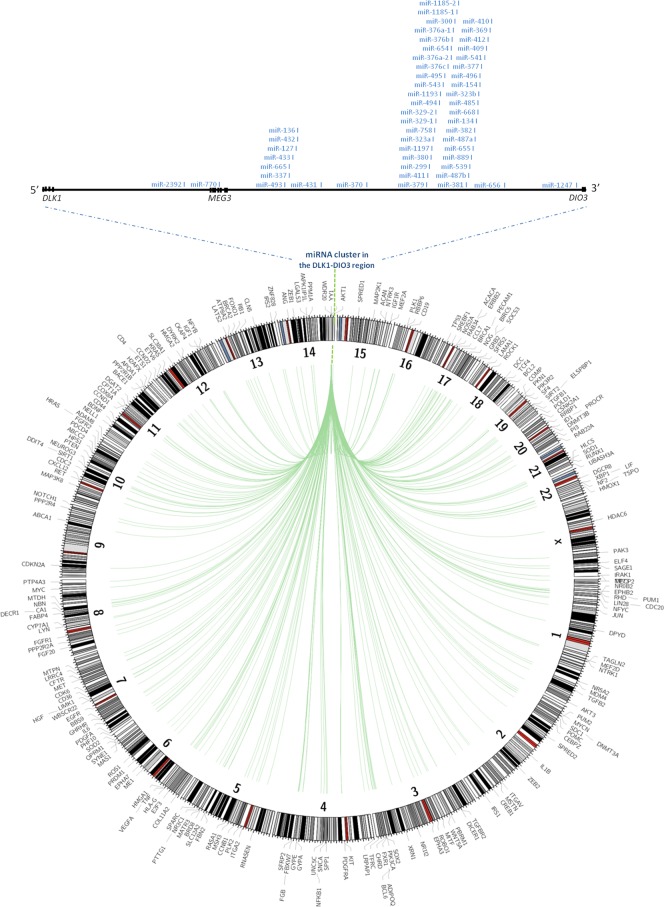
Validated targets of miRNAs contained in the *DLK1-DIO3* cluster Plot showing with position of validated miRNA targets in the whole human genome. The validated targets are connected to the miRNAs within the *DLK1-DIO3* region on chromosome 14 by green lines.

To study the functional relevance of the *DLK1-DIO3* miRNA cluster in the regulation of validated mRNAs as a potential tumor suppressor, we used the miRWalk algorithm [27]. A total of 538 genes were found to be validated targets of the miRNAs included in this cluster. These targets were located on all chromosomes except the Y chromosome (Figure 7). Several miRNAs were complementary with more than one validated gene, and some targets were regulated by more than one miRNA. We classified validated targets by gene ontology (GO) molecular function and biological processes using the PANTHER program. The main GO molecular functions were binding (GO:0005488) (38.9%), catalytic activity (GO:0003824) (27.4%), nucleic acid binding transcription factor activity (GO:0001071) (13.5%), and receptor activity (GO:0004872) (10.4%). The primary binding types were protein binding (GO:0005515) and nucleic acid binding (GO:0003676). In the case of catalytic activity, we primarily found activities for hydrolase (GO:0016787), transferase (GO:0016740) and enzyme regulators (GO:0007154). Validated targets displayed six main biological processes: metabolic processes (GO:0008152) (21.2%), such as cell communication (GO:0030234) and cell cycle (GO:0007049); cellular process (GO:0009987) (20.2%), including system development (GO:0048731), anatomical structure and morphogenesis (GO:0009653) and death (GO:0016265); developmental processes (GO:0032502) (12.4%); response to stimulus (GO:0050896) (5.6%); and apoptotic processes (GO:0006915) (5.3%) (Supplementary Figure S1). Finally, we found that 26 out of 538 validated genes (*AXIN2, BRCA1, CCNB1, CCND2, CDKN1A, CDKN2A, CSNK2A1, FOXO1, GRB2, HDAC6, NFKB1, NTRK3, PPP2R4, PPP2R1B, PPP2R2A, PTEN, RARA, RASA1, RB1, SOCS3, TGFB1, TGFB2, TGFBR2, TNF, TP53, and TP73*) were targets of miRNAs included in the *DLK1-DIO3* cluster, which have been proposed to have a functional role as tumor suppressors (Supplementary Table S2).

## DISCUSSION

In the present study we firstly identified characteristic methylation pattern of the *DLK1-DIO3* cluster in human lung cancer, as compared, to lung parenchyma. Specifically, we identified that all components of the afore-mentioned cluster are hypomethylated, with the exception of *DIO3* that is hypermethylated. Importantly this specific methylation signature of the imprinted cluster is restricted to smoking induced lung cancer since it is absent in lung tumors from never smokers. In addition, this locus was more aberrantly regulated in SCC, typically exposed to heavier smoking history, than in lung adenocarcinoma. In a murine lung carcinoma model with mutated *KRAS*, it has been observed that the *DLK1-DIO3* miRNAs cluster on chromosome on 12qF1 was substantially overexpressed, suggesting a role as an oncogenic driver of lung cancer in these circumstances [26]. Those findings are consistent with our results, particularly considering KRAS is the most frequently mutated proto-oncogenes among smokers with lung carcinoma [28, 29].

Imprinted genes are particularly more susceptible to epigenetic regulatory changes due to having one specifically silenced allele [6]. Therefore, monoallelic changes in methylation status may have deleterious consequences. The *DLK1-DIO3* cluster includes protein-coding genes, lncRNAs, miRNAs, SNORDs and pseudogenes [21]. *DLK1, RTL1* and *DIO3* are expressed only from the paternal allele. However, large and small noncoding RNA genes and pseudogenes come from the maternal allele [30]. Our data showed that the paternally derived *DLK1* and *RTL1* genes are hypomethylated and that the *DIO3* gene is hypermethylated. *DLK1* has been reported aberrantly expressed in various types of tumors, such as hepatocellular carcinoma, glioma, renal cell carcinoma and lung cancer [31–34]. In addition, Li et al. recently identified in an *in vitro* lung model that overexpression of *DLK1* enhanced extracellular matrix invasion by Notch signaling in a dependent and independent manner [23]. In the case of the *RTL1* gene, it has been proposed to act as an oncogene in hepatocarcinoma. *In vivo RTL1* overexpression in the liver contributed to tumor growth [24]. Accordingly, the hypomethylation of these genes can play an important role in the pathogenesis of lung cancer. The hypermethylation of *DIO3* has been reported in hematologic neoplasms [35]. We also identified higher levels of global DNA methylation in lung cancer versus normal lung tissue. Moreover, the *MEG3* lncRNA has been described as a tumor suppressor; *MEG3* levels were significantly downregulated in patients with NSCLC [36]. However, our study showed that the methylation level was significantly higher in human lung cancer relative to non-tumoral tissue. These differences could be due to the differences between the subjects recruited for each study. Lu *et al*. reported lower *MEG3* expression in patients with an advanced pathological stage [36], while in our study, almost 80% of patients were at clinical stage I or II. Otherwise, the loss of expression of this tumor suppressor could also be due to micro-deletion at the *MEG3-DMR* locus or deletion of a transcription factor-binding site on the *MEG3* promoter [37, 38]. In the case of *MEG8* and *MEG9* lncRNAs, their functions in cancer remain unknown.

In our study, we observed an important pattern of DNA hypomethylation in two SNORD families and two miRNA clusters in patients with lung cancer. In addition, we visualized less CpG and strong transcription in this region. MicroRNAs are post-transcriptional modulators of many biological processes. The dysregulation of their physiological roles may contribute to the initiation and progression of cancer [39]. Along with *miRNAs*, other types of small regulatory non-protein-coding RNAs were detected, such as small nucleolar RNAs (snoRNAs). It has been reported that alterations in snoRNA expression levels may lead to various diseases, including cancer [40, 41]. Valleron *et al*. found that several variants of SNORDs of the *DLK1-DIO3* cluster were overexpressed in patients with acute promyelocytic leukemia (APL) [42]. In addition, it has been shown that *in vitro SNORD114-1* overexpression promoted cell growth by cell cycle modulation [42]. The same results have been obtained in a large cohort of of acute promyelocytic leukemia (APL) patients, suggesting that increased expression of *SNORD114-3* is a good biomarker of APL [43]. In our study, we identified that the increased expression of *SNORD114-3* was only statistically significant in the SCC group although a tendency was observed in the lung adenocarcinoma group. The *DLK1-DIO3* cluster contains also 53 miRNAs. The deregulation of some of them has been associated with enhanced tumorigenicity [22]. MicroRNA-154, miR-379 and miR-409 were highly expressed in metastatic prostate cancer cells [44]. MicroRNA-127 was overexpressed in the serum of patients with esophageal squamous cell carcinoma [45]. Haller *et al.* revealed the deregulation of *miRNA-329, miR-370, miR-376c* and *miR409* expression based on the location of the tumor in gastrointestinal stromal tumors (GISTs); higher expression occurred in intestinal compared to gastric tumors [46]. Indeed, it has even been proposed that *miR-376c* can promote cell proliferation and invasion [47, 48]. Recently, miRNAs of the *DLK1-DIO3* cluster have been identified as being upregulated in a mouse model of lung adenocarcinoma [26]. The results obtained in a mouse model are in line with those obtained from the patient cohort of our study. We found a pattern of DNA hypomethylation in the *DLK1-DIO3* microRNA cluster in current and former smoker patients with lung cancer. This profile was representative of both SCC and lung adenocarcinoma histological subtypes. On the other hand, it has been found that hepatocellular carcinoma patients with overexpression of the *DLK1-DIO3* miRNA cluster had significantly poorer overall survival [49]. In addition, 3 miRNAs of the *DLK1-DIO3* cluster have been associated with poor survival in patients with surgically resected lung adenocarcinoma [50]. These data warrant a validation of the *DLK1-DIO3* cluster methylation as a prognostic tool in a prospective cohort study.

We identified experimentally validated targets and predicted their molecular function. We found that they may be involved in catalytic activity and the binding of nucleic acids to transcription factors. The biological process categories were mostly metabolic, cellular, developmental, response to stimulus and apoptotic processes. In addition, we identified 26 validated targets, including *AXIN2, BRCA1, CCNB1, CCND2, CDKN1A, CDKN2A, CSNK2A1, FOXO1, GRB2, HDAC6, NFKB1, NTRK3, PPP2R4, PPP2R1B, PPP2R2A, PTEN, RARA, RASA1, RB1, SOCS3, TGFB1, TGFB2, TGFBR2, TNF, TP53, and TP73*, which can act as tumor suppressors. Thus, the hypomethylation of the *DLK1-DIO3* miRNA cluster may lead to its activation, promoting the downregulation of these relevant tumor suppressors in lung cancer.

In conclusion, we found the *DLK1-DIO3* cluster to be hypomethylated in current and former smoker patients with NSCLC. This pattern suggests that aberrant expression may contribute to tumorigenesis in the lung. Moreover, given that some components of the *DLK1-DIO3* cluster may be responsible of the regulation of tumor suppressors in lung cancer, they could have great potential as novel biomarkers and therapeutic targets. The present analysis suggests that future studies on this topic are warranted.

## MATERIALS AND METHODS

### Patients and clinical specimens

The present study was performed in 70 subjects fromVirgen del Rocio University Hospital (Seville). The samples were divided into two cohorts. A first group of samples, from 47 patients who had undergone surgical resection for clinical early stage NSCLC. During the surgical procedure, the tumor and matched non-tumoral tissue samples were collected from all patients and then immediately snap-frozen to −80°C until further use. The clinical features of patients with NSCLC are summarized in Supplementary Table S3. A second group from 23 subjects was used as control cohort. The control cohort without lung cancer was constituted of COPD patients and non-COPD subjects. A description of this cohort can be found on Supplementary Table S4. A written consent form was obtained from all participants. The study protocol and the use of human samples were approved by the Ethical Committee of the Virgen del Rocio University Hospital.

### DNA samples

Genomic DNA was extracted from tumor and matched non-tumoral tissue samples by the QIAamp DNA mini kit (QIAGEN, Valencia, CA, USA). DNA was quantified using the QuantiFluor dsDNA system (Promega, Madison, WI, USA) according to the manufacturers’ instructions.

### Illumina 450 K methylation

The Illumina Infinium Human Methylation 450 BeadChip (Illumina Inc., San Diego, CA) was used to interrogate 485,000 methylation sites across the genome per sample at single-nucleotide resolution. It covers 96% of the CpG islands, with additional coverage in island shores and the flanking regions. We treated 500 ng of DNA with sodium bisulfate using the EZ DNA Methylation™ Kit and cleaned the DNA with the ZR-96 DNA Clean-up Kit™ (EZ DNA, Zymo Research, Irvine, CA) before standard Illumina amplification, hybridization, and imaging steps. The resulting intensity files were analyzed with Illumina's GenomeStudio, which generated β-scores (i.e., the proportion of total signal from the methylation-specific probe or color channel).

### Methylome data processing

Methylome data were processed using the RnBeads R package [51]. After a quality check, the probe median intensity was normalized with the SWAN method [52] and converted to beta values. The probes were tested for differential methylation with the limma method, a linear model followed by empirical Bayes methods for the comparisons of interest [53]. The Benjamini–Hochberg method was used to adjust the *p*-values and ensure that the false discovery rate (FDR) was lower than 0.05. The CpG status (hypo- versus hyper-methylated) and CpG chromosomal location were realized using the Circos data visualization software [54]. DNA methylation data were visualized by the Wash U Epigenome Browser [55]. Validated mRNA targets of miRNAs by previous research located in the *DLK1-DIO3* cluster were identified with the miRWalk algorithm [27]. The molecular function and proposed biological process of experimentally verified mRNAs were determined using the PANTHER program [56].

### Validation of DNA methylation status

Smoking-related samples from lung cancer patients in TCGA database (https://tcga-data.nci.nih.gov) were selected to interrogate the *DLK1-DIO3* cluster methylation status. Their characteristics are summarized in Table S5. Differences on the DLK1-DIO3 methylation profile between tumor and normal tissues from current and former smoker lung cancer patients were identified with a method previously described [57]. Non-smoking habit of lung cancer patients in TCGA database were often incomplete. For this reason, 454 bisulfite sequencing (Roche, Mannheim, Germany) was used to validate the DNA methylation status of *DLK1*, *miR-889* and *SNORD114-9* [58]. Primer sequences are listed in Supplementary table S6 and the genomic features of regions selected for 454 bisulfite sequencing are represented in Supplementary Figure S2.

### Expression levels of some *DLK1-DIO3* cluster components

Expression of some randomly selected *DLK1-DIO3* cluster components (*DIO3*, *SNORD113-5*, *SNORD113-7*, *SNORD114-9*, and *miR-889*) was analyzed by qPCR. For genes, 100 ng/μL of total RNA was converted into cDNA by the reverse transcriptase reaction which was performed by sequential incubation at 25°C for 10 min, 37°C for 2 h and 85°C for 5 min. For the miR-889 expression levels, 2 ng/μL of total RNA was converted into cDNA by the reverse transcriptase reaction, which was performed by sequential incubation at 16°C for 30 min, 42°C for 30 min and 85°C for 5 min. The PCR reaction mixture was initially incubated at 95°C for 10 min, followed by 40 cycles of 95°C for 15 seconds and 60°C for 60 seconds. The expression of *miR-889* was normalized to the expression of *RNU48* and *B2M* for the normalization of different genes. Gene expression data of tumor versus normal tissue were compared by a t-test. The results were processed and analyzed as previously described [59, 60].

## SUPPLEMENTARY MATERIALS FIGURES AND TABLES








