# Supplementary material for: Impact of DLK1-DIO3 imprinted cluster hypomethylation in smoker patients with lung cancer
Source: Oncotarget. 2016 Jul 15;9(4):4395–410. doi: 10.18632/oncotarget.10611 (PMC5796982; doi:10.18632/oncotarget.10611)
Supplement: Supplementary file 1 [file oncotarget-09-4395-s001.pdf]

Impact of DLK1-DIO3 imprinted cluster hypomethylation in SMOKER patients with lung cancer

SUPPLEMENTARY FIGURES AND TABLES

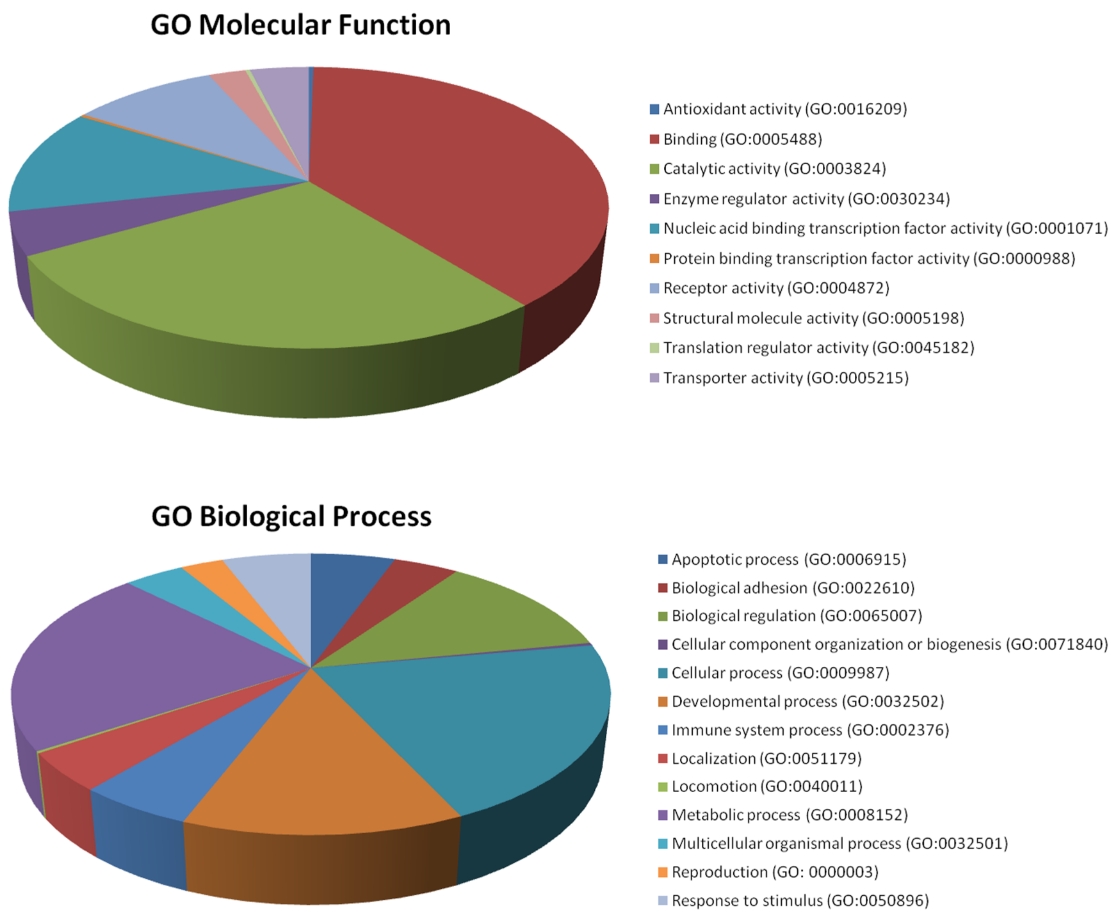

Supplementary Figure S1: Classification of validated targets by gene ontology (GO) molecular function and biological processes. Summary of molecular functions and biological processes for validated target genes of miRNAs included in the *DLK1-DIO3* cluster. Approximately 39% of the genes were annotated to binding and more than 20% of the genes were involved in metabolic processes.

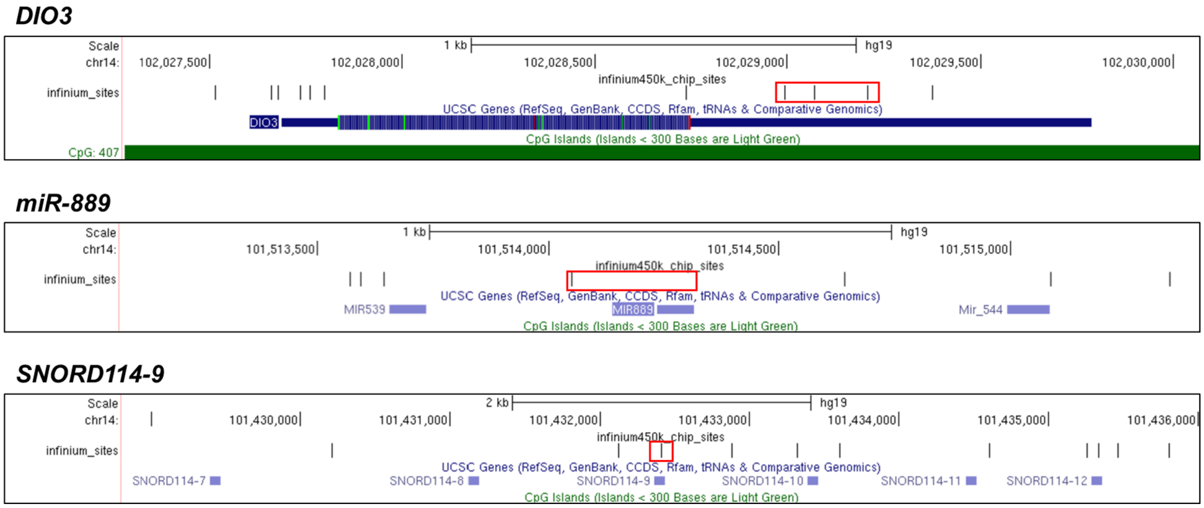

Supplementary Figure S2: Genomic features of *DIO3, miR-889* and *SNORD114-9*. These schemes indicate the CpG islands present on the region. The red squares designate the genomic regions selected for 454 bisulfite sequencing.

Supplementary Table S1: The methylation levels in lung cancer versus mou-tumoral tissue.

See Supplementary File 1

Supplementary Table S2: Validated tumor suppressors of miRNAs included in the DLK1-DIO3 cluster.

See Supplementary File 2

Supplementary Table S3: Characteristics of the study cohort

	Patients (N=47)
**Gender**	
Male	76.6 (36)
Female	23.4 (11)
**Age (years)**	67 [60-73]
**Smoking status**	
Smokers	40.4 (19)
Ex-smokers	44.7 (21)
Non-smoker	14.9 (7)
**Packs/year**	41.0 [20.0-65.7]
**Histology**	
Lung adenocarcinoma	57.4 (27)
Squamous cell carcinoma	42.6 (20)
**Staging**	
Stage I	40.5 (19)
Stage II	38.3 (18)
Stage III-IV	21.2 (10)
**Subjects with COPD**	42.6 (20)

Continuous variables are expressed as the median [interquartile range (IQR)] and categorical variables are expressed as the number of cases (%).

Supplementary Table S4: Description of control cohort

	Controls (N=23)
**Gender**	
Male	87.0 (20)
Female	13.0 (3)
**Age (years)**	35 [21-62]
**Smoking status**	
Smokers	30.4 (7)
Ex-smokers	21.7 (5)
Non-smoker	47.8 (11)
**Packs/year**	2.0 [2.0-20.0]
**Subjects with COPD**	17.4 (4)

Continuous variables are expressed as the median [interquartile range (IQR)] and categorical variables are expressed as the number of cases (%).

Supplementary Table S5: Characteristics of the TCGA cohort.

See Supplementary File 3

Supplementary Table S6: List of primer sequences used for 454 bisulfite sequencing

**454 Bisulfite Sequencing**	
**Primers**	**Sequences**
F *DIO3*	TGTTTGGTTTATTGGAAATTTTTTT
R *DIO3*	AACAACAAAAAACCCCCTCTC
F *mir-889*	TATTAGGGATTTGTATTGGTTATG
R *mir-889*	TTTAAATACTAAAACAATAATTA
F *SNORD114-9*	TATTTAGGTTATTTTTGGGGTTTTT
R *SNORD114-9*	AACCATAAACAAATAAATTCTATATCTCTA
