# Supplementary material for: Impact of DLK1-DIO3 imprinted cluster hypomethylation in smoker patients with lung cancer
Source: Oncotarget. 2016 Jul 15;9(4):4395–410. doi: 10.18632/oncotarget.10611 (PMC5796982; doi:10.18632/oncotarget.10611)
Supplement: Supplementary file 2 [file oncotarget-09-4395-s002.pptx]

## Slide 1
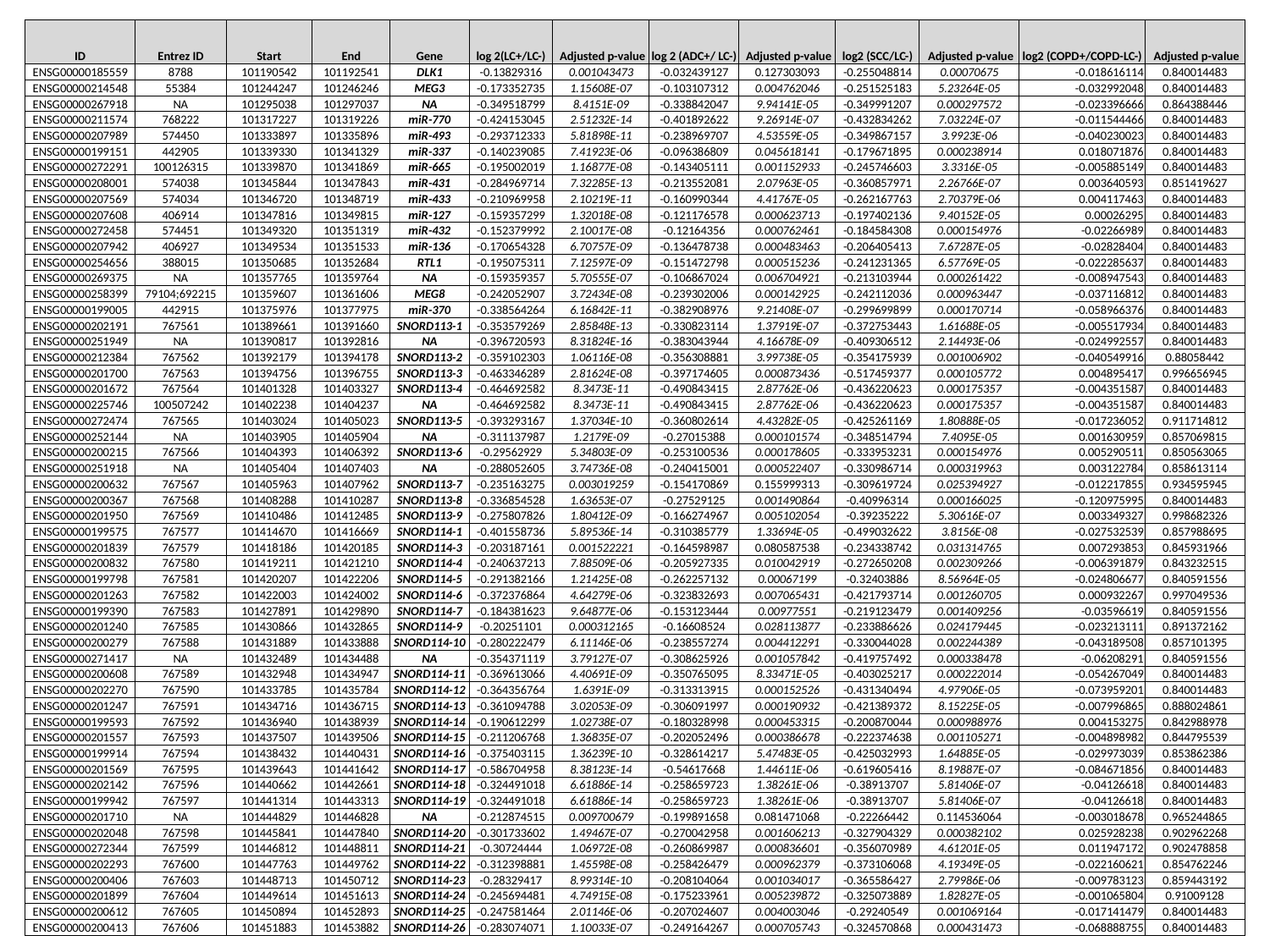

| ID | Entrez ID | Start | End | Gene | log 2(LC+/LC-) | Adjusted p-value | log 2 (ADC+/ LC-) | Adjusted p-value | log2 (SCC/LC-) | Adjusted p-value | log2 (COPD+/COPD-LC-) | Adjusted p-value |
| --- | --- | --- | --- | --- | --- | --- | --- | --- | --- | --- | --- | --- |
| ENSG00000185559 | 8788 | 101190542 | 101192541 | DLK1 | -0.13829316 | 0.001043473 | -0.032439127 | 0.127303093 | -0.255048814 | 0.00070675 | -0.018616114 | 0.840014483 |
| ENSG00000214548 | 55384 | 101244247 | 101246246 | MEG3 | -0.173352735 | 1.15608E-07 | -0.103107312 | 0.004762046 | -0.251525183 | 5.23264E-05 | -0.032992048 | 0.840014483 |
| ENSG00000267918 | NA | 101295038 | 101297037 | NA | -0.349518799 | 8.4151E-09 | -0.338842047 | 9.94141E-05 | -0.349991207 | 0.000297572 | -0.023396666 | 0.864388446 |
| ENSG00000211574 | 768222 | 101317227 | 101319226 | miR-770 | -0.424153045 | 2.51232E-14 | -0.401892622 | 9.26914E-07 | -0.432834262 | 7.03224E-07 | -0.011544466 | 0.840014483 |
| ENSG00000207989 | 574450 | 101333897 | 101335896 | miR-493 | -0.293712333 | 5.81898E-11 | -0.238969707 | 4.53559E-05 | -0.349867157 | 3.9923E-06 | -0.040230023 | 0.840014483 |
| ENSG00000199151 | 442905 | 101339330 | 101341329 | miR-337 | -0.140239085 | 7.41923E-06 | -0.096386809 | 0.045618141 | -0.179671895 | 0.000238914 | 0.018071876 | 0.840014483 |
| ENSG00000272291 | 100126315 | 101339870 | 101341869 | miR-665 | -0.195002019 | 1.16877E-08 | -0.143405111 | 0.001152933 | -0.245746603 | 3.3316E-05 | -0.005885149 | 0.840014483 |
| ENSG00000208001 | 574038 | 101345844 | 101347843 | miR-431 | -0.284969714 | 7.32285E-13 | -0.213552081 | 2.07963E-05 | -0.360857971 | 2.26766E-07 | 0.003640593 | 0.851419627 |
| ENSG00000207569 | 574034 | 101346720 | 101348719 | miR-433 | -0.210969958 | 2.10219E-11 | -0.160990344 | 4.41767E-05 | -0.262167763 | 2.70379E-06 | 0.004117463 | 0.840014483 |
| ENSG00000207608 | 406914 | 101347816 | 101349815 | miR-127 | -0.159357299 | 1.32018E-08 | -0.121176578 | 0.000623713 | -0.197402136 | 9.40152E-05 | 0.00026295 | 0.840014483 |
| ENSG00000272458 | 574451 | 101349320 | 101351319 | miR-432 | -0.152379992 | 2.10017E-08 | -0.12164356 | 0.000762461 | -0.184584308 | 0.000154976 | -0.02266989 | 0.840014483 |
| ENSG00000207942 | 406927 | 101349534 | 101351533 | miR-136 | -0.170654328 | 6.70757E-09 | -0.136478738 | 0.000483463 | -0.206405413 | 7.67287E-05 | -0.02828404 | 0.840014483 |
| ENSG00000254656 | 388015 | 101350685 | 101352684 | RTL1 | -0.195075311 | 7.12597E-09 | -0.151472798 | 0.000515236 | -0.241231365 | 6.57769E-05 | -0.022285637 | 0.840014483 |
| ENSG00000269375 | NA | 101357765 | 101359764 | NA | -0.159359357 | 5.70555E-07 | -0.106867024 | 0.006704921 | -0.213103944 | 0.000261422 | -0.008947543 | 0.840014483 |
| ENSG00000258399 | 79104;692215 | 101359607 | 101361606 | MEG8 | -0.242052907 | 3.72434E-08 | -0.239302006 | 0.000142925 | -0.242112036 | 0.000963447 | -0.037116812 | 0.840014483 |
| ENSG00000199005 | 442915 | 101375976 | 101377975 | miR-370 | -0.338564264 | 6.16842E-11 | -0.382908976 | 9.21408E-07 | -0.299699899 | 0.000170714 | -0.058966376 | 0.840014483 |
| ENSG00000202191 | 767561 | 101389661 | 101391660 | SNORD113-1 | -0.353579269 | 2.85848E-13 | -0.330823114 | 1.37919E-07 | -0.372753443 | 1.61688E-05 | -0.005517934 | 0.840014483 |
| ENSG00000251949 | NA | 101390817 | 101392816 | NA | -0.396720593 | 8.31824E-16 | -0.383043944 | 4.16678E-09 | -0.409306512 | 2.14493E-06 | -0.024992557 | 0.840014483 |
| ENSG00000212384 | 767562 | 101392179 | 101394178 | SNORD113-2 | -0.359102303 | 1.06116E-08 | -0.356308881 | 3.99738E-05 | -0.354175939 | 0.001006902 | -0.040549916 | 0.88058442 |
| ENSG00000201700 | 767563 | 101394756 | 101396755 | SNORD113-3 | -0.463346289 | 2.81624E-08 | -0.397174605 | 0.000873436 | -0.517459377 | 0.000105772 | 0.004895417 | 0.996656945 |
| ENSG00000201672 | 767564 | 101401328 | 101403327 | SNORD113-4 | -0.464692582 | 8.3473E-11 | -0.490843415 | 2.87762E-06 | -0.436220623 | 0.000175357 | -0.004351587 | 0.840014483 |
| ENSG00000225746 | 100507242 | 101402238 | 101404237 | NA | -0.464692582 | 8.3473E-11 | -0.490843415 | 2.87762E-06 | -0.436220623 | 0.000175357 | -0.004351587 | 0.840014483 |
| ENSG00000272474 | 767565 | 101403024 | 101405023 | SNORD113-5 | -0.393293167 | 1.37034E-10 | -0.360802614 | 4.43282E-05 | -0.425261169 | 1.80888E-05 | -0.017236052 | 0.911714812 |
| ENSG00000252144 | NA | 101403905 | 101405904 | NA | -0.311137987 | 1.2179E-09 | -0.27015388 | 0.000101574 | -0.348514794 | 7.4095E-05 | 0.001630959 | 0.857069815 |
| ENSG00000200215 | 767566 | 101404393 | 101406392 | SNORD113-6 | -0.29562929 | 5.34803E-09 | -0.253100536 | 0.000178605 | -0.333953231 | 0.000154976 | 0.005290511 | 0.850563065 |
| ENSG00000251918 | NA | 101405404 | 101407403 | NA | -0.288052605 | 3.74736E-08 | -0.240415001 | 0.000522407 | -0.330986714 | 0.000319963 | 0.003122784 | 0.858613114 |
| ENSG00000200632 | 767567 | 101405963 | 101407962 | SNORD113-7 | -0.235163275 | 0.003019259 | -0.154170869 | 0.155999313 | -0.309619724 | 0.025394927 | -0.012217855 | 0.934595945 |
| ENSG00000200367 | 767568 | 101408288 | 101410287 | SNORD113-8 | -0.336854528 | 1.63653E-07 | -0.27529125 | 0.001490864 | -0.40996314 | 0.000166025 | -0.120975995 | 0.840014483 |
| ENSG00000201950 | 767569 | 101410486 | 101412485 | SNORD113-9 | -0.275807826 | 1.80412E-09 | -0.166274967 | 0.005102054 | -0.39235222 | 5.30616E-07 | 0.003349327 | 0.998682326 |
| ENSG00000199575 | 767577 | 101414670 | 101416669 | SNORD114-1 | -0.401558736 | 5.89536E-14 | -0.310385779 | 1.33694E-05 | -0.499032622 | 3.8156E-08 | -0.027532539 | 0.857988695 |
| ENSG00000201839 | 767579 | 101418186 | 101420185 | SNORD114-3 | -0.203187161 | 0.001522221 | -0.164598987 | 0.080587538 | -0.234338742 | 0.031314765 | 0.007293853 | 0.845931966 |
| ENSG00000200832 | 767580 | 101419211 | 101421210 | SNORD114-4 | -0.240637213 | 7.88509E-06 | -0.205927335 | 0.010042919 | -0.272650208 | 0.002309266 | -0.006391879 | 0.843232515 |
| ENSG00000199798 | 767581 | 101420207 | 101422206 | SNORD114-5 | -0.291382166 | 1.21425E-08 | -0.262257132 | 0.00067199 | -0.32403886 | 8.56964E-05 | -0.024806677 | 0.840591556 |
| ENSG00000201263 | 767582 | 101422003 | 101424002 | SNORD114-6 | -0.372376864 | 4.64279E-06 | -0.323832693 | 0.007065431 | -0.421793714 | 0.001260705 | 0.000932267 | 0.997049536 |
| ENSG00000199390 | 767583 | 101427891 | 101429890 | SNORD114-7 | -0.184381623 | 9.64877E-06 | -0.153123444 | 0.00977551 | -0.219123479 | 0.001409256 | -0.03596619 | 0.840591556 |
| ENSG00000201240 | 767585 | 101430866 | 101432865 | SNORD114-9 | -0.20251101 | 0.000312165 | -0.16608524 | 0.028113877 | -0.233886626 | 0.024179445 | -0.023213111 | 0.891372162 |
| ENSG00000200279 | 767588 | 101431889 | 101433888 | SNORD114-10 | -0.280222479 | 6.11146E-06 | -0.238557274 | 0.004412291 | -0.330044028 | 0.002244389 | -0.043189508 | 0.857101395 |
| ENSG00000271417 | NA | 101432489 | 101434488 | NA | -0.354371119 | 3.79127E-07 | -0.308625926 | 0.001057842 | -0.419757492 | 0.000338478 | -0.06208291 | 0.840591556 |
| ENSG00000200608 | 767589 | 101432948 | 101434947 | SNORD114-11 | -0.369613066 | 4.40691E-09 | -0.350765095 | 8.33471E-05 | -0.403025217 | 0.000222014 | -0.054267049 | 0.840014483 |
| ENSG00000202270 | 767590 | 101433785 | 101435784 | SNORD114-12 | -0.364356764 | 1.6391E-09 | -0.313313915 | 0.000152526 | -0.431340494 | 4.97906E-05 | -0.073959201 | 0.840014483 |
| ENSG00000201247 | 767591 | 101434716 | 101436715 | SNORD114-13 | -0.361094788 | 3.02053E-09 | -0.306091997 | 0.000190932 | -0.421389372 | 8.15225E-05 | -0.007996865 | 0.888024861 |
| ENSG00000199593 | 767592 | 101436940 | 101438939 | SNORD114-14 | -0.190612299 | 1.02738E-07 | -0.180328998 | 0.000453315 | -0.200870044 | 0.000988976 | 0.004153275 | 0.842988978 |
| ENSG00000201557 | 767593 | 101437507 | 101439506 | SNORD114-15 | -0.211206768 | 1.36835E-07 | -0.202052496 | 0.000386678 | -0.222374638 | 0.001105271 | -0.004898982 | 0.844795539 |
| ENSG00000199914 | 767594 | 101438432 | 101440431 | SNORD114-16 | -0.375403115 | 1.36239E-10 | -0.328614217 | 5.47483E-05 | -0.425032993 | 1.64885E-05 | -0.029973039 | 0.853862386 |
| ENSG00000201569 | 767595 | 101439643 | 101441642 | SNORD114-17 | -0.586704958 | 8.38123E-14 | -0.54617668 | 1.44611E-06 | -0.619605416 | 8.19887E-07 | -0.084671856 | 0.840014483 |
| ENSG00000202142 | 767596 | 101440662 | 101442661 | SNORD114-18 | -0.324491018 | 6.61886E-14 | -0.258659723 | 1.38261E-06 | -0.38913707 | 5.81406E-07 | -0.04126618 | 0.840014483 |
| ENSG00000199942 | 767597 | 101441314 | 101443313 | SNORD114-19 | -0.324491018 | 6.61886E-14 | -0.258659723 | 1.38261E-06 | -0.38913707 | 5.81406E-07 | -0.04126618 | 0.840014483 |
| ENSG00000201710 | NA | 101444829 | 101446828 | NA | -0.212874515 | 0.009700679 | -0.199891658 | 0.081471068 | -0.22266442 | 0.114536064 | -0.003018678 | 0.965244865 |
| ENSG00000202048 | 767598 | 101445841 | 101447840 | SNORD114-20 | -0.301733602 | 1.49467E-07 | -0.270042958 | 0.001606213 | -0.327904329 | 0.000382102 | 0.025928238 | 0.902962268 |
| ENSG00000272344 | 767599 | 101446812 | 101448811 | SNORD114-21 | -0.30724444 | 1.06972E-08 | -0.260869987 | 0.000836601 | -0.356070989 | 4.61201E-05 | 0.011947172 | 0.902478858 |
| ENSG00000202293 | 767600 | 101447763 | 101449762 | SNORD114-22 | -0.312398881 | 1.45598E-08 | -0.258426479 | 0.000962379 | -0.373106068 | 4.19349E-05 | -0.022160621 | 0.854762246 |
| ENSG00000200406 | 767603 | 101448713 | 101450712 | SNORD114-23 | -0.28329417 | 8.99314E-10 | -0.208104064 | 0.001034017 | -0.365586427 | 2.79986E-06 | -0.009783123 | 0.859443192 |
| ENSG00000201899 | 767604 | 101449614 | 101451613 | SNORD114-24 | -0.245694481 | 4.74915E-08 | -0.175233961 | 0.005239872 | -0.325073889 | 1.82827E-05 | -0.001065804 | 0.91009128 |
| ENSG00000200612 | 767605 | 101450894 | 101452893 | SNORD114-25 | -0.247581464 | 2.01146E-06 | -0.207024607 | 0.004003046 | -0.29240549 | 0.001069164 | -0.017141479 | 0.840014483 |
| ENSG00000200413 | 767606 | 101451883 | 101453882 | SNORD114-26 | -0.283074071 | 1.10033E-07 | -0.249164267 | 0.000705743 | -0.324570868 | 0.000431473 | -0.068888755 | 0.840014483 |

## Slide 2
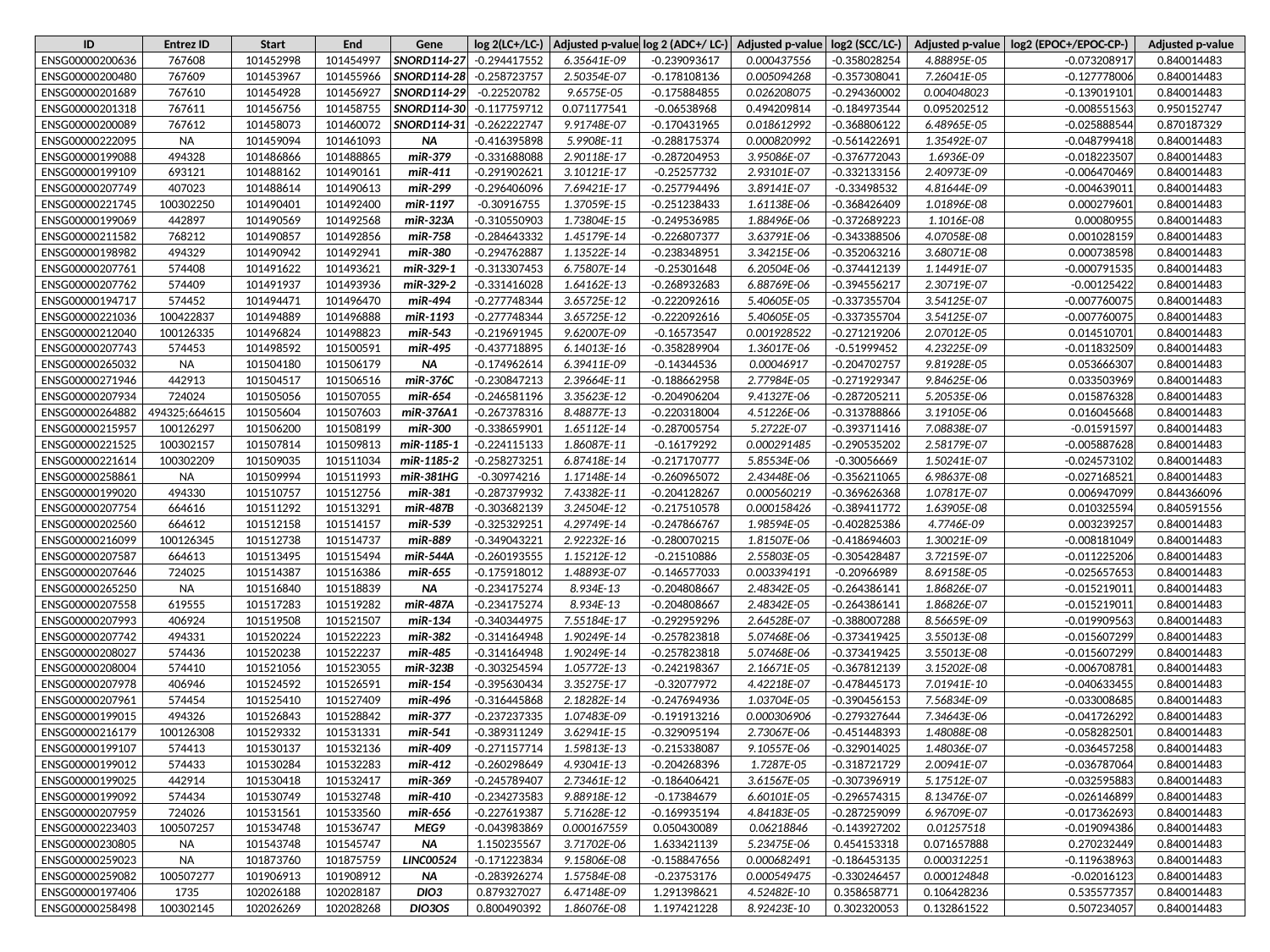

| ID | Entrez ID | Start | End | Gene | log 2(LC+/LC-) | Adjusted p-value | log 2 (ADC+/ LC-) | Adjusted p-value | log2 (SCC/LC-) | Adjusted p-value | log2 (EPOC+/EPOC-CP-) | Adjusted p-value |
| --- | --- | --- | --- | --- | --- | --- | --- | --- | --- | --- | --- | --- |
| ENSG00000200636 | 767608 | 101452998 | 101454997 | SNORD114-27 | -0.294417552 | 6.35641E-09 | -0.239093617 | 0.000437556 | -0.358028254 | 4.88895E-05 | -0.073208917 | 0.840014483 |
| ENSG00000200480 | 767609 | 101453967 | 101455966 | SNORD114-28 | -0.258723757 | 2.50354E-07 | -0.178108136 | 0.005094268 | -0.357308041 | 7.26041E-05 | -0.127778006 | 0.840014483 |
| ENSG00000201689 | 767610 | 101454928 | 101456927 | SNORD114-29 | -0.22520782 | 9.6575E-05 | -0.175884855 | 0.026208075 | -0.294360002 | 0.004048023 | -0.139019101 | 0.840014483 |
| ENSG00000201318 | 767611 | 101456756 | 101458755 | SNORD114-30 | -0.117759712 | 0.071177541 | -0.06538968 | 0.494209814 | -0.184973544 | 0.095202512 | -0.008551563 | 0.950152747 |
| ENSG00000200089 | 767612 | 101458073 | 101460072 | SNORD114-31 | -0.262222747 | 9.91748E-07 | -0.170431965 | 0.018612992 | -0.368806122 | 6.48965E-05 | -0.025888544 | 0.870187329 |
| ENSG00000222095 | NA | 101459094 | 101461093 | NA | -0.416395898 | 5.9908E-11 | -0.288175374 | 0.000820992 | -0.561422691 | 1.35492E-07 | -0.048799418 | 0.840014483 |
| ENSG00000199088 | 494328 | 101486866 | 101488865 | miR-379 | -0.331688088 | 2.90118E-17 | -0.287204953 | 3.95086E-07 | -0.376772043 | 1.6936E-09 | -0.018223507 | 0.840014483 |
| ENSG00000199109 | 693121 | 101488162 | 101490161 | miR-411 | -0.291902621 | 3.10121E-17 | -0.25257732 | 2.93101E-07 | -0.332133156 | 2.40973E-09 | -0.006470469 | 0.840014483 |
| ENSG00000207749 | 407023 | 101488614 | 101490613 | miR-299 | -0.296406096 | 7.69421E-17 | -0.257794496 | 3.89141E-07 | -0.33498532 | 4.81644E-09 | -0.004639011 | 0.840014483 |
| ENSG00000221745 | 100302250 | 101490401 | 101492400 | miR-1197 | -0.30916755 | 1.37059E-15 | -0.251238433 | 1.61138E-06 | -0.368426409 | 1.01896E-08 | 0.000279601 | 0.840014483 |
| ENSG00000199069 | 442897 | 101490569 | 101492568 | miR-323A | -0.310550903 | 1.73804E-15 | -0.249536985 | 1.88496E-06 | -0.372689223 | 1.1016E-08 | 0.00080955 | 0.840014483 |
| ENSG00000211582 | 768212 | 101490857 | 101492856 | miR-758 | -0.284643332 | 1.45179E-14 | -0.226807377 | 3.63791E-06 | -0.343388506 | 4.07058E-08 | 0.001028159 | 0.840014483 |
| ENSG00000198982 | 494329 | 101490942 | 101492941 | miR-380 | -0.294762887 | 1.13522E-14 | -0.238348951 | 3.34215E-06 | -0.352063216 | 3.68071E-08 | 0.000738598 | 0.840014483 |
| ENSG00000207761 | 574408 | 101491622 | 101493621 | miR-329-1 | -0.313307453 | 6.75807E-14 | -0.25301648 | 6.20504E-06 | -0.374412139 | 1.14491E-07 | -0.000791535 | 0.840014483 |
| ENSG00000207762 | 574409 | 101491937 | 101493936 | miR-329-2 | -0.331416028 | 1.64162E-13 | -0.268932683 | 6.88769E-06 | -0.394556217 | 2.30719E-07 | -0.00125422 | 0.840014483 |
| ENSG00000194717 | 574452 | 101494471 | 101496470 | miR-494 | -0.277748344 | 3.65725E-12 | -0.222092616 | 5.40605E-05 | -0.337355704 | 3.54125E-07 | -0.007760075 | 0.840014483 |
| ENSG00000221036 | 100422837 | 101494889 | 101496888 | miR-1193 | -0.277748344 | 3.65725E-12 | -0.222092616 | 5.40605E-05 | -0.337355704 | 3.54125E-07 | -0.007760075 | 0.840014483 |
| ENSG00000212040 | 100126335 | 101496824 | 101498823 | miR-543 | -0.219691945 | 9.62007E-09 | -0.16573547 | 0.001928522 | -0.271219206 | 2.07012E-05 | 0.014510701 | 0.840014483 |
| ENSG00000207743 | 574453 | 101498592 | 101500591 | miR-495 | -0.437718895 | 6.14013E-16 | -0.358289904 | 1.36017E-06 | -0.51999452 | 4.23225E-09 | -0.011832509 | 0.840014483 |
| ENSG00000265032 | NA | 101504180 | 101506179 | NA | -0.174962614 | 6.39411E-09 | -0.14344536 | 0.00046917 | -0.204702757 | 9.81928E-05 | 0.053666307 | 0.840014483 |
| ENSG00000271946 | 442913 | 101504517 | 101506516 | miR-376C | -0.230847213 | 2.39664E-11 | -0.188662958 | 2.77984E-05 | -0.271929347 | 9.84625E-06 | 0.033503969 | 0.840014483 |
| ENSG00000207934 | 724024 | 101505056 | 101507055 | miR-654 | -0.246581196 | 3.35623E-12 | -0.204906204 | 9.41327E-06 | -0.287205211 | 5.20535E-06 | 0.015876328 | 0.840014483 |
| ENSG00000264882 | 494325;664615 | 101505604 | 101507603 | miR-376A1 | -0.267378316 | 8.48877E-13 | -0.220318004 | 4.51226E-06 | -0.313788866 | 3.19105E-06 | 0.016045668 | 0.840014483 |
| ENSG00000215957 | 100126297 | 101506200 | 101508199 | miR-300 | -0.338659901 | 1.65112E-14 | -0.287005754 | 5.2722E-07 | -0.393711416 | 7.08838E-07 | -0.01591597 | 0.840014483 |
| ENSG00000221525 | 100302157 | 101507814 | 101509813 | miR-1185-1 | -0.224115133 | 1.86087E-11 | -0.16179292 | 0.000291485 | -0.290535202 | 2.58179E-07 | -0.005887628 | 0.840014483 |
| ENSG00000221614 | 100302209 | 101509035 | 101511034 | miR-1185-2 | -0.258273251 | 6.87418E-14 | -0.217170777 | 5.85534E-06 | -0.30056669 | 1.50241E-07 | -0.024573102 | 0.840014483 |
| ENSG00000258861 | NA | 101509994 | 101511993 | miR-381HG | -0.30974216 | 1.17148E-14 | -0.260965072 | 2.43448E-06 | -0.356211065 | 6.98637E-08 | -0.027168521 | 0.840014483 |
| ENSG00000199020 | 494330 | 101510757 | 101512756 | miR-381 | -0.287379932 | 7.43382E-11 | -0.204128267 | 0.000560219 | -0.369626368 | 1.07817E-07 | 0.006947099 | 0.844366096 |
| ENSG00000207754 | 664616 | 101511292 | 101513291 | miR-487B | -0.303682139 | 3.24504E-12 | -0.217510578 | 0.000158426 | -0.389411772 | 1.63905E-08 | 0.010325594 | 0.840591556 |
| ENSG00000202560 | 664612 | 101512158 | 101514157 | miR-539 | -0.325329251 | 4.29749E-14 | -0.247866767 | 1.98594E-05 | -0.402825386 | 4.7746E-09 | 0.003239257 | 0.840014483 |
| ENSG00000216099 | 100126345 | 101512738 | 101514737 | miR-889 | -0.349043221 | 2.92232E-16 | -0.280070215 | 1.81507E-06 | -0.418694603 | 1.30021E-09 | -0.008181049 | 0.840014483 |
| ENSG00000207587 | 664613 | 101513495 | 101515494 | miR-544A | -0.260193555 | 1.15212E-12 | -0.21510886 | 2.55803E-05 | -0.305428487 | 3.72159E-07 | -0.011225206 | 0.840014483 |
| ENSG00000207646 | 724025 | 101514387 | 101516386 | miR-655 | -0.175918012 | 1.48893E-07 | -0.146577033 | 0.003394191 | -0.20966989 | 8.69158E-05 | -0.025657653 | 0.840014483 |
| ENSG00000265250 | NA | 101516840 | 101518839 | NA | -0.234175274 | 8.934E-13 | -0.204808667 | 2.48342E-05 | -0.264386141 | 1.86826E-07 | -0.015219011 | 0.840014483 |
| ENSG00000207558 | 619555 | 101517283 | 101519282 | miR-487A | -0.234175274 | 8.934E-13 | -0.204808667 | 2.48342E-05 | -0.264386141 | 1.86826E-07 | -0.015219011 | 0.840014483 |
| ENSG00000207993 | 406924 | 101519508 | 101521507 | miR-134 | -0.340344975 | 7.55184E-17 | -0.292959296 | 2.64528E-07 | -0.388007288 | 8.56659E-09 | -0.019909563 | 0.840014483 |
| ENSG00000207742 | 494331 | 101520224 | 101522223 | miR-382 | -0.314164948 | 1.90249E-14 | -0.257823818 | 5.07468E-06 | -0.373419425 | 3.55013E-08 | -0.015607299 | 0.840014483 |
| ENSG00000208027 | 574436 | 101520238 | 101522237 | miR-485 | -0.314164948 | 1.90249E-14 | -0.257823818 | 5.07468E-06 | -0.373419425 | 3.55013E-08 | -0.015607299 | 0.840014483 |
| ENSG00000208004 | 574410 | 101521056 | 101523055 | miR-323B | -0.303254594 | 1.05772E-13 | -0.242198367 | 2.16671E-05 | -0.367812139 | 3.15202E-08 | -0.006708781 | 0.840014483 |
| ENSG00000207978 | 406946 | 101524592 | 101526591 | miR-154 | -0.395630434 | 3.35275E-17 | -0.32077972 | 4.42218E-07 | -0.478445173 | 7.01941E-10 | -0.040633455 | 0.840014483 |
| ENSG00000207961 | 574454 | 101525410 | 101527409 | miR-496 | -0.316445868 | 2.18282E-14 | -0.247694936 | 1.03704E-05 | -0.390456153 | 7.56834E-09 | -0.033008685 | 0.840014483 |
| ENSG00000199015 | 494326 | 101526843 | 101528842 | miR-377 | -0.237237335 | 1.07483E-09 | -0.191913216 | 0.000306906 | -0.279327644 | 7.34643E-06 | -0.041726292 | 0.840014483 |
| ENSG00000216179 | 100126308 | 101529332 | 101531331 | miR-541 | -0.389311249 | 3.62941E-15 | -0.329095194 | 2.73067E-06 | -0.451448393 | 1.48088E-08 | -0.058282501 | 0.840014483 |
| ENSG00000199107 | 574413 | 101530137 | 101532136 | miR-409 | -0.271157714 | 1.59813E-13 | -0.215338087 | 9.10557E-06 | -0.329014025 | 1.48036E-07 | -0.036457258 | 0.840014483 |
| ENSG00000199012 | 574433 | 101530284 | 101532283 | miR-412 | -0.260298649 | 4.93041E-13 | -0.204268396 | 1.7287E-05 | -0.318721729 | 2.00941E-07 | -0.036787064 | 0.840014483 |
| ENSG00000199025 | 442914 | 101530418 | 101532417 | miR-369 | -0.245789407 | 2.73461E-12 | -0.186406421 | 3.61567E-05 | -0.307396919 | 5.17512E-07 | -0.032595883 | 0.840014483 |
| ENSG00000199092 | 574434 | 101530749 | 101532748 | miR-410 | -0.234273583 | 9.88918E-12 | -0.17384679 | 6.60101E-05 | -0.296574315 | 8.13476E-07 | -0.026146899 | 0.840014483 |
| ENSG00000207959 | 724026 | 101531561 | 101533560 | miR-656 | -0.227619387 | 5.71628E-12 | -0.169935194 | 4.84183E-05 | -0.287259099 | 6.96709E-07 | -0.017362693 | 0.840014483 |
| ENSG00000223403 | 100507257 | 101534748 | 101536747 | MEG9 | -0.043983869 | 0.000167559 | 0.050430089 | 0.06218846 | -0.143927202 | 0.01257518 | -0.019094386 | 0.840014483 |
| ENSG00000230805 | NA | 101543748 | 101545747 | NA | 1.150235567 | 3.71702E-06 | 1.633421139 | 5.23475E-06 | 0.454153318 | 0.071657888 | 0.270232449 | 0.840014483 |
| ENSG00000259023 | NA | 101873760 | 101875759 | LINC00524 | -0.171223834 | 9.15806E-08 | -0.158847656 | 0.000682491 | -0.186453135 | 0.000312251 | -0.119638963 | 0.840014483 |
| ENSG00000259082 | 100507277 | 101906913 | 101908912 | NA | -0.283926274 | 1.57584E-08 | -0.23753176 | 0.000549475 | -0.330246457 | 0.000124848 | -0.02016123 | 0.840014483 |
| ENSG00000197406 | 1735 | 102026188 | 102028187 | DIO3 | 0.879327027 | 6.47148E-09 | 1.291398621 | 4.52482E-10 | 0.358658771 | 0.106428236 | 0.535577357 | 0.840014483 |
| ENSG00000258498 | 100302145 | 102026269 | 102028268 | DIO3OS | 0.800490392 | 1.86076E-08 | 1.197421228 | 8.92423E-10 | 0.302320053 | 0.132861522 | 0.507234057 | 0.840014483 |
