# Supplementary material for: Impact of DLK1-DIO3 imprinted cluster hypomethylation in smoker patients with lung cancer
Source: Oncotarget. 2016 Jul 15;9(4):4395–410. doi: 10.18632/oncotarget.10611 (PMC5796982; doi:10.18632/oncotarget.10611)
Supplement: Supplementary file 3 [file oncotarget-09-4395-s003.docx]

**Supplementary table S2.** Validated tumor suppressors of miRNAs included in the *DLK1-DIO3* cluster.

| Tumor Suppressor Gene | Validated miRNAs | References |
| --- | --- | --- |
| *AXIN2* | *miR-299* | [1] |
| *BRCA1* | *miR-154, miR-369, miR-370 y miR-381* | [2-3] |
| *CCNB1* | *miR-494* | [4] |
| *CCND2* | *miR-154* | [5] |
| *CDKN1A* | *miR-299, miR-337, miR-377 y miR-654* | [6-8] |
| *CDKN2A* | *miR-127, miR-299, miR-379 y miR-410* | [9-10] |
| *CSNK2A1* | *miR-337* | [7] |
| *FOXO1* | *miR-299* | [1] |
| *GRB2* | *miR-433* | [11] |
| *HDAC6* | *miR-433* | [12-13] |
| *NFKB1* | *miR-410 y miR-433* | [10, 14] |
| *NTRK3* | *miR-485* | [15] |
| *PPP2R4* | *miR-136* | [16] |
| *PPP2R1B* | *miR-134* | [17] |
| *PPP2R2A* | *miR-136* | [16] |
| *PTEN* | *miR-136 y miR-494* | [18-21] |
| *RARA* | *miR-370* | [22] |
| *RASA1* | *miR-127* | [23] |
| *RB1* | *miR136 y miR-410* | [10, 18] |
| *SOCS3* | *miR-337* | [24] |
| *TGFB1* | *miR-369, miR-370 y miR-382* | [25-26] |
| *TGFB2* | *miR-369 y miR-370* | [25] |
| *TGFBR2* | *miR-337* | [27] |
| *TNF* | *miR-369 y miR-377* | [28-29] |
| *TP53* | *miR-134, miR-337, miR-380 y miR-410* | [7, 10, 30-31] |
| *TP73* | *miR-410* | [30] |

**REFERENCES of Supplementary table S4**

1. Liu X, He M, Hou Y, Liang B, Zhao L, Ma S and Yu Y. Expression profiles of microRNAs and their target genes in papillary thyroid carcinoma. Oncol Rep. 2013; 29(4):1415-1420.

2. Shen J, DiCioccio R, Odunsi K, Lele SB and Zhao H. Novel genetic variants in miR-191 gene and familial ovarian cancer. BMC Cancer. 2010; 10:47.

3. Pastrello C, Polesel J, Della Puppa L, Viel A and Maestro R. Association between hsa-mir-146a genotype and tumor age-of-onset in BRCA1/BRCA2-negative familial breast and ovarian cancer patients. Carcinogenesis. 2010; 31(12):2124-2126.

4. Yamanaka S, Campbell NR, An F, Kuo SC, Potter JJ, Mezey E, Maitra A and Selaru FM. Coordinated effects of microRNA-494 induce G(2)/M arrest in human cholangiocarcinoma. Cell Cycle. 2012; 11(14):2729-2738.

5. Wang W, Peng B, Wang D, Ma X, Jiang D, Zhao J and Yu L. Human tumor microRNA signatures derived from large-scale oligonucleotide microarray datasets. Int J Cancer. 2011; 129(7):1624-1634.

6. Wu S, Huang S, Ding J, Zhao Y, Liang L, Liu T, Zhan R and He X. Multiple microRNAs modulate p21Cip1/Waf1 expression by directly targeting its 3' untranslated region. Oncogene. 2010; 29(15):2302-2308.

7. Kim SY, Lee YH and Bae YS. MiR-186, miR-216b, miR-337-3p, and miR-760 cooperatively induce cellular senescence by targeting alpha subunit of protein kinase CKII in human colorectal cancer cells. Biochem Biophys Res Commun. 2012; 429(3-4):173-179.

8. Wang Q, Wang Y, Minto AW, Wang J, Shi Q, Li X and Quigg RJ. MicroRNA-377 is up-regulated and can lead to increased fibronectin production in diabetic nephropathy. FASEB J. 2008; 22(12):4126-4135.

9. Guled M, Lahti L, Lindholm PM, Salmenkivi K, Bagwan I, Nicholson AG and Knuutila S. CDKN2A, NF2, and JUN are dysregulated among other genes by miRNAs in malignant mesothelioma -A miRNA microarray analysis. Genes Chromosomes Cancer. 2009; 48(7):615-623.

10. Chien WW, Domenech C, Catallo R, Kaddar T, Magaud JP, Salles G and Ffrench M. Cyclin-dependent kinase 1 expression is inhibited by p16(INK4a) at the post-transcriptional level through the microRNA pathway. Oncogene. 2011; 30(16):1880-1891.

11. Luo H, Zhang H, Zhang Z, Zhang X, Ning B, Guo J, Nie N, Liu B and Wu X. Down-regulated miR-9 and miR-433 in human gastric carcinoma. J Exp Clin Cancer Res. 2009; 28:82.

12. Laloo B, Maurel M, Jalvy-Delvaille S, Sagliocco F and Grosset CF. Analysis of post-transcriptional regulation using the FunREG method. Biochem Soc Trans. 2010; 38(6):1608-1614.

13. Simon D, Laloo B, Barillot M, Barnetche T, Blanchard C, Rooryck C, Marche M, Burgelin I, Coupry I, Chassaing N, Gilbert-Dussardier B, Lacombe D, Grosset C and Arveiler B. A mutation in the 3'-UTR of the HDAC6 gene abolishing the post-transcriptional regulation mediated by hsa-miR-433 is linked to a new form of dominant X-linked chondrodysplasia. Hum Mol Genet. 2010; 19(10):2015-2027.

14. Estep M, Armistead D, Hossain N, Elarainy H, Goodman Z, Baranova A, Chandhoke V and Younossi ZM. Differential expression of miRNAs in the visceral adipose tissue of patients with non-alcoholic fatty liver disease. Aliment Pharmacol Ther. 2010; 32(3):487-497.

15. Mian C, Pennelli G, Fassan M, Balistreri M, Barollo S, Cavedon E, Galuppini F, Pizzi M, Vianello F, Pelizzo MR, Girelli ME, Rugge M and Opocher G. MicroRNA profiles in familial and sporadic medullary thyroid carcinoma: preliminary relationships with RET status and outcome. Thyroid. 2012; 22(9):890-896.

16. Liu X, Sempere LF, Ouyang H, Memoli VA, Andrew AS, Luo Y, Demidenko E, Korc M, Shi W, Preis M, Dragnev KH, Li H, Direnzo J, Bak M, Freemantle SJ, Kauppinen S, et al. MicroRNA-31 functions as an oncogenic microRNA in mouse and human lung cancer cells by repressing specific tumor suppressors. J Clin Invest. 2010; 120(4):1298-1309.

17. Hamano R, Miyata H, Yamasaki M, Kurokawa Y, Hara J, Moon JH, Nakajima K, Takiguchi S, Fujiwara Y, Mori M and Doki Y. Overexpression of miR-200c induces chemoresistance in esophageal cancers mediated through activation of the Akt signaling pathway. Clin Cancer Res. 2011; 17(9):3029-3038.

18. Lee DY, Jeyapalan Z, Fang L, Yang J, Zhang Y, Yee AY, Li M, Du WW, Shatseva T and Yang BB. Expression of versican 3'-untranslated region modulates endogenous microRNA functions. PLoS One. 2010; 5(10):e13599.

19. Wang X, Zhang X, Ren XP, Chen J, Liu H, Yang J, Medvedovic M, Hu Z and Fan GC. MicroRNA-494 targeting both proapoptotic and antiapoptotic proteins protects against ischemia/reperfusion-induced cardiac injury. Circulation. 2010; 122(13):1308-1318.

20. Liu Y, Lai L, Chen Q, Song Y, Xu S, Ma F, Wang X, Wang J, Yu H, Cao X and Wang Q. MicroRNA-494 is required for the accumulation and functions of tumor-expanded myeloid-derived suppressor cells via targeting of PTEN. J Immunol. 2012; 188(11):5500-5510.

21. Liu L, Jiang Y, Zhang H, Greenlee AR and Han Z. Overexpressed miR-494 down-regulates PTEN gene expression in cells transformed by anti-benzo(a)pyrene-trans-7,8-dihydrodiol-9,10-epoxide. Life Sci. 2010; 86(5-6):192-198.

22. Liu DZ, Ander BP, Tian Y, Stamova B, Jickling GC, Davis RR and Sharp FR. Integrated analysis of mRNA and microRNA expression in mature neurons, neural progenitor cells and neuroblastoma cells. Gene. 2012; 495(2):120-127.

23. Lim PK, Bliss SA, Patel SA, Taborga M, Dave MA, Gregory LA, Greco SJ, Bryan M, Patel PS and Rameshwar P. Gap junction-mediated import of microRNA from bone marrow stromal cells can elicit cell cycle quiescence in breast cancer cells. Cancer Res. 2011; 71(5):1550-1560.

24. Bakre A, Mitchell P, Coleman JK, Jones LP, Saavedra G, Teng M, Tompkins SM and Tripp RA. Respiratory syncytial virus modifies microRNAs regulating host genes that affect virus replication. J Gen Virol. 2012; 93(Pt 11):2346-2356.

25. Castilla MA, Moreno-Bueno G, Romero-Perez L, Van De Vijver K, Biscuola M, Lopez-Garcia MA, Prat J, Matias-Guiu X, Cano A, Oliva E and Palacios J. Micro-RNA signature of the epithelial-mesenchymal transition in endometrial carcinosarcoma. J Pathol. 2011; 223(1):72-80.

26. Kriegel AJ, Fang Y, Liu Y, Tian Z, Mladinov D, Matus IR, Ding X, Greene AS and Liang M. MicroRNA-target pairs in human renal epithelial cells treated with transforming growth factor beta 1: a novel role of miR-382. Nucleic Acids Res. 2010; 38(22):8338-8347.

27. Zhong N, Sun J, Min Z, Zhao W, Zhang R, Wang W, Tian J, Tian L, Ma J, Li D, Han Y and Lu S. MicroRNA-337 is associated with chondrogenesis through regulating TGFBR2 expression. Osteoarthritis Cartilage. 2012; 20(6):593-602.

28. Vasudevan S, Tong Y and Steitz JA. Switching from repression to activation: microRNAs can up-regulate translation. Science. 2007; 318(5858):1931-1934.

29. Delic D, Dkhil M, Al-Quraishy S and Wunderlich F. Hepatic miRNA expression reprogrammed by Plasmodium chabaudi malaria. Parasitol Res. 2011; 108(5):1111-1121.

30. Boominathan L. The tumor suppressors p53, p63, and p73 are regulators of microRNA processing complex. PLoS One. 2010; 5(5):e10615.

31. Swarbrick A, Woods SL, Shaw A, Balakrishnan A, Phua Y, Nguyen A, Chanthery Y, Lim L, Ashton LJ, Judson RL, Huskey N, Blelloch R, Haber M, Norris MD, Lengyel P, Hackett CS, et al. miR-380-5p represses p53 to control cellular survival and is associated with poor outcome in MYCN-amplified neuroblastoma. Nat Med. 2010; 16(10):1134-1140.
